# Aetiology and incidence of diarrhoea requiring hospitalisation in children under 5 years of age in 28 low-income and middle-income countries: findings from the Global Pediatric Diarrhea Surveillance network

**DOI:** 10.1136/bmjgh-2022-009548

**Published:** 2022-09-05

**Authors:** Adam L Cohen, James A Platts-Mills, Tomoka Nakamura, Darwin J Operario, Sébastien Antoni, Jason M Mwenda, Goitom Weldegebriel, Gloria Rey-Benito, Lucia H de Oliveira, Claudia Ortiz, Danni S Daniels, Dovile Videbaek, Simarjit Singh, Emmanuel Njambe, Mohamed Sharifuzzaman, Varja Grabovac, Batmunkh Nyambat, Josephine Logronio, George Armah, Francis E Dennis, Mapaseka L Seheri, Nokululeko Magagula, Jeffrey Mphahlele, Tulio M Fumian, Irene T A Maciel, Jose Paulo Gagliardi Leite, Matthew D Esona, Michael D Bowen, Elena Samoilovich, Galina Semeiko, Dilip Abraham, Sidhartha Giri, Ira Praharaj, Gagandeep Kang, Sarah Thomas, Julie Bines, Na Liu, Hmwe H Kyu, Matthew Doxey, Elizabeth T Rogawski McQuade, Timothy L McMurry, Jie Liu, Eric R Houpt, Jacqueline E Tate, Umesh D Parashar, Fatima Serhan

**Affiliations:** 1 National Center for Immunization and Respiratory Diseases, Influenza Division, CDC, Atlanta, Georgia, USA; 2 Division of Infectious Diseases and International Health, University of Virginia, Charlottesville, Virginia, USA; 3 World Health Organization, Geneve, Switzerland; 4 World Health Organization Regional Office for Africa, Brazzaville, Congo; 5 World Health Organization Regional Office for the Americas, Washington, DC, USA; 6 World Health Organization Regional Office for Europe, Copenhagen, Denmark; 7 World Health Organization Regional Office for South-East Asia, New Delhi, India; 8 World Health Organization Regional Office for the Western Pacific, Manila, Philippines; 9 University of Ghana Noguchi Memorial Institute for Medical Research, Accra, Ghana; 10 Sefako Makgatho Health Sciences University, Pretoria, South Africa; 11 Institute Oswaldo Cruz, Rio de Janeiro, Brazil; 12 Divison of Viral Diseases, Centers for Disease Control and Prevention, Atlanta, Georgia, USA; 13 Republican Research and Practical Center for Epidemiology and Microbiology, Minsk, Belarus; 14 Christian Medical College Vellore, Vellore, India; 15 Indian Council of Medical Research Regiona lMedical Research Centre, Bhubaneswar, India; 16 Murdoch Children's Research Institute, Parkville, Victoria, Australia; 17 Chinese Center for Disease Control and Prevention, Beijing, China; 18 Institute for Health Metrics and Evaluation, Seattle, Washington, USA; 19 Department of Epidemiology, Emory University, Atlanta, Georgia, USA; 20 Qingdao University, Qingdao, Shandong, China

**Keywords:** public health, epidemiology, infections, diseases, disorders, injuries, PCR, child health

## Abstract

**Introduction:**

Diarrhoea remains a leading cause of child morbidity and mortality. Systematically collected and analysed data on the aetiology of hospitalised diarrhoea in low-income and middle-income countries are needed to prioritise interventions.

**Methods:**

We established the Global Pediatric Diarrhea Surveillance network, in which children under 5 years hospitalised with diarrhoea were enrolled at 33 sentinel surveillance hospitals in 28 low-income and middle-income countries. Randomly selected stool specimens were tested by quantitative PCR for 16 causes of diarrhoea. We estimated pathogen-specific attributable burdens of diarrhoeal hospitalisations and deaths. We incorporated country-level incidence to estimate the number of pathogen-specific deaths on a global scale.

**Results:**

During 2017–2018, 29 502 diarrhoea hospitalisations were enrolled, of which 5465 were randomly selected and tested. Rotavirus was the leading cause of diarrhoea requiring hospitalisation (attributable fraction (AF) 33.3%; 95% CI 27.7 to 40.3), followed by *Shigella* (9.7%; 95% CI 7.7 to 11.6), norovirus (6.5%; 95% CI 5.4 to 7.6) and adenovirus 40/41 (5.5%; 95% CI 4.4 to 6.7). Rotavirus was the leading cause of hospitalised diarrhoea in all regions except the Americas, where the leading aetiologies were *Shigella* (19.2%; 95% CI 11.4 to 28.1) and norovirus (22.2%; 95% CI 17.5 to 27.9) in Central and South America, respectively. The proportion of hospitalisations attributable to rotavirus was approximately 50% lower in sites that had introduced rotavirus vaccine (AF 20.8%; 95% CI 18.0 to 24.1) compared with sites that had not (42.1%; 95% CI 33.2 to 53.4). Globally, we estimated 208 009 annual rotavirus-attributable deaths (95% CI 169 561 to 259 216), 62 853 *Shigella*-attributable deaths (95% CI 48 656 to 78 805), 36 922 adenovirus 40/41-attributable deaths (95% CI 28 469 to 46 672) and 35 914 norovirus-attributable deaths (95% CI 27 258 to 46 516).

**Conclusions:**

Despite the substantial impact of rotavirus vaccine introduction, rotavirus remained the leading cause of paediatric diarrhoea hospitalisations. Improving the efficacy and coverage of rotavirus vaccination and prioritising interventions against *Shigella*, norovirus and adenovirus could further reduce diarrhoea morbidity and mortality.

**What is already known on this topic**

- Aetiological studies using quantitative molecular diagnostics have primarily studied children in the community or those presenting to care, of which a minority are hospitalised.
- Those studies have suggested that several pathogens other than rotavirus, including *Shigella*, had a higher or similar burden, especially after rotavirus vaccine introduction.

**What this study adds**

- This study used standardised surveillance, laboratory and analysis protocols to define the aetiology of hospitalised paediatric diarrhoea using quantitative molecular diagnostics from a large, globally representative set of low-income and middle-income countries, including countries and geographies that were not well represented in the published literature.
- Despite a substantial impact of rotavirus vaccination, rotavirus remained the leading cause of diarrhoea requiring hospitalisation. *Shigella*, norovirus and adenovirus 40/41 also had a high burden of disease, with some important geographic heterogeneity.

**How this study might affect research, practice or policy**

- Despite increasing use of rotavirus vaccine globally, countries should expand its use and increase coverage, and strategies to improve rotavirus vaccine efficacy should be pursued.
- Interventions targeting *Shigella*, norovirus and enteric group adenoviruses should be prioritised while recognising that the impacts of such interventions would be expected to vary by region.

## Introduction

Despite steady declines in deaths from paediatric diarrhoea over the past three decades, diarrhoea remains a leading cause of death and disease in children less than 5 years of age, causing roughly half a million childhood deaths each year, predominantly in sub-Saharan Africa and south and southeast Asia.^1^ The introduction of rotavirus vaccines into national immunisation programmes decreased severe rotavirus diarrhoea in children by approximately 40% in low-income and middle-income countries (LMICs) from 2008 to 2016.^2^ Ongoing monitoring and surveillance of paediatric diarrhoea and its causes are needed to identify diarrhoea prevention and control strategies and guide the prioritisation and development of new vaccines for enteric pathogens.^3 4^

Diarrhoea requiring hospitalisation represents a consistently severe phenotype of this highly morbid syndrome. This proxy is explicitly used by groups that estimate the global burden of diarrhoeal deaths as diarrhoea severe enough to require hospitalisation should have a similar aetiological profile.^1 5 6^ Quantitative PCR (qPCR) has emerged as the preferred diagnostic approach for aetiological studies of diarrhoea,^7^ but most estimates using these diagnostics are from studies of primarily non-hospitalised diarrhoea.^8–10^ Thus, global estimates of aetiology-specific paediatric diarrhoea hospitalisations and mortality are driven by older studies of hospitalised diarrhoea that used a broad range of non-molecular diagnostics^5 11^ and studies of non-hospitalised diarrhoea with adjustments to approximate the expected aetiology in hospitalised diarrhoea.^1^ There is a dearth of globally representative, direct data on the aetiology of diarrhoea in hospitalised children using quantitative molecular diagnostics to inform global burden estimates and to monitor longitudinal trends in diarrhoea aetiology in the era of rotavirus vaccine use, particularly in LMICs where the burden is highest.^12–14^

To ensure high-quality data on rotavirus from high-burden countries with limited surveillance and laboratory capacity, the WHO has coordinated the Global Rotavirus Surveillance Network (GRSN) since 2008, enrolling children with acute watery diarrhoea and testing for rotavirus at national laboratories and regional reference laboratories (RRLs).^15 16^ In 2015, a retrospective pilot analysis of a limited number of non-randomly selected diarrhoeal specimens from children hospitalised for acute watery diarrhoea in 16 countries, predominantly from sub-Saharan Africa, trialled the use of a TaqMan Array card (TAC) qPCR assay system to broaden the list of aetiologies monitored in cases of diarrhoea enrolled in GRSN.^17^ In the current study, we leveraged this existing capacity to create the Global Pediatric Diarrhea Surveillance (GPDS) network, expanding the case definition to all hospitalised diarrhoea and including a globally representative selection of LMICs, with a primary goal of defining and monitoring trends in the causative enteropathogens in all children hospitalised with diarrhoea in these settings.

## Methods

### Study design and sentinel site selection

We performed a cross-sectional study of children hospitalised with diarrhoea. Sites were chosen from the broader GRSN to be broadly geographically representative. Sites were required to have a history of uninterrupted surveillance, with enrolment of a minimum of 100 cases of hospitalised diarrhoea in children less than 5 years of age per year. The identified surveillance sites expanded their case definition from acute, watery diarrhoea only to prospectively enrol all children admitted for diarrhoea, regardless of duration or the presence of blood.^18 19^ Thirty-three GRSN sentinel surveillance sites in 28 globally representative countries with a history of uninterrupted surveillance and high-quality data collection participated in prospective, active surveillance of all children less than 5 years of age hospitalised with diarrhoea (online supplemental table 1). The surveillance sites in Côte d’Ivoire, Ethiopia, Mauritius, Moldova and Zimbabwe aggregated patients from two or three surveillance hospitals within the same catchment area. Three GPDS countries had surveillance sites in more than one catchment area: China (three sites), India (three sites) and Zambia (two sites). Each WHO Region had three to six countries participate in GPDS, except the African region, which had 10, and the Eastern Mediterranean region, which was not included. We considered rotavirus vaccines to be introduced at each site if rotavirus vaccination had been added to the immunisation programme at the country or regional level by the end of 2017. The Strengthening the Reporting of Observational Studies in Epidemiology (STROBE) checklist for observational studies is included in the online supplemental materials.

10.1136/bmjgh-2022-009548.supp1 Supplementary data

10.1136/bmjgh-2022-009548.supp2 Supplementary data

### Case definition and enrolment

The inclusion criteria for enrolment in GPDS was any child aged 0–59 months hospitalised with diarrhoea. Diarrhoea was defined as three or more loose stools in a 24-hour period. Children transferred from another hospital or who developed diarrhoea during their hospitalisation were excluded. Caregivers of children enrolled in GPDS were asked about demographic and clinical characteristics of the cases, including reported duration of symptoms and reported or observed presence of blood in stool; medical records were also reviewed. Acute diarrhoea was defined as an episode with a duration prior to enrolment of fewer than 14 days, while longer episodes were considered persistent. Bloody diarrhoea was identified by caregiver report of blood in stool. Dehydration was noted and in a subset of cases, dehydration severity was estimated using the WHO severity scale.^20^ Deidentified data were used to maintain the anonymity of patients.

### Specimen collection, selection, extraction and qPCR testing

Stool specimen collection was attempted from all enrolled cases. Specimens were stored at a maximum temperature of −20°C and at −80°C when possible. All specimens were tested for rotavirus using a commercial ELISA as previously described.^2^ To be eligible for random selection for qPCR testing, cases had to have a stool sample collected, and data on the duration of diarrhoea and presence of blood in the stool had to be available. From each surveillance site, 25 cases were randomly selected for qPCR testing for each 3 month quarter starting 1 January 2017 and ending 31 December 2018; the quarters included varied by site (online supplemental table 1). All participating sites contributed data and samples from both 2017 and 2018 except for Bolivia, which was added in 2018. Whole stool aliquots were then shipped with ice packs (or dry ice when possible) to an RRL and stored at −80°C prior to nucleic acid extraction.

All RRLs participated in site assessments and on-site training as well as annual external quality assurance and quality control exercises.^19^ Total nucleic acid extraction was performed as previously described with the Qiagen QIAamp Fast DNA Stool Mini kit (Qiagen, Hilden, Germany) using a modified protocol involving bead beating, with nucleic acid samples subsequently stored at −80°C prior to testing.^17^ All samples were spiked with Phocine herpes virus and MS2 phage to be used as external controls for DNA and RNA, respectively, and an extraction blank was included in each extraction batch to monitor for contamination. Sample testing using custom-designed TaqMan Array Cards (Thermo Fisher, Waltham, Massachusetts, USA) was performed as previously described.^17^ The array card included qPCR assays for 16 enteric pathogens that were associated with diarrhoea in two prior multisite studies that incorporated diarrhoeal cases and non-diarrhoeal controls (online supplemental table 2).^9 10^

Raw data analysis was carried out at each of the RRLs with fluorescence thresholds standardised for each qPCR assay between all eight RRLs using software appropriate to each laboratory’s instrument. Detections with a quantification cycle (Cq) value less than 35 were considered positive. Negative results were valid only when the corresponding external control was positive, while positive results were valid only when the corresponding extraction blank was negative for the target. We excluded data flagged by the PCR software.

### Statistical analysis

Because samples were selected for qPCR testing by stratified random selection for each 3 month quarter, we applied inverse probability of selection weights such that cases selected for testing were representative of all enrolled cases and appropriately captured seasonal variation in diarrhoea aetiology. Specifically, we fit a generalised linear model with selection for qPCR testing as the outcome and site, 3 month quarter and an interaction between site and 3 month quarter as the covariates. Weights were then calculated as the inverse of the model-predicted probability. These weights were applied to all study estimates that used the subset of tested samples, including clinical characteristics, pathogen prevalence, attributable fractions (AFs) and incidence of hospitalised diarrhoea and attributable deaths.

Asymptomatic identification of enteropathogens is common in these settings, especially when using molecular testing methods, and non-diarrhoeal controls were not collected in the present study. Therefore, we attributed diarrhoea to specific enteropathogens based on pathogen quantity using models developed from qPCR reanalyses of two multisite aetiologic studies of diarrhoea that incorporated non-diarrhoeal controls, the GEMS case–control study and the MAL-ED birth cohort study.^8–10^ Model development for those studies has previously been described in detail.^9 10^ Briefly, using a conditional logistic regression model for GEMS and a generalised linear mixed-effects model with a random effect for each individual for MAL-ED, a model was fit for each pathogen to describe the association between pathogen quantity and diarrhoea, with a random slope for site to allow for variation in the strength of association between pathogen quantity and diarrhoea. The MAL-ED model was additionally adjusted for sex, age and TAC card batch. Pathogen quantity was defined as the log10 increase in pathogen quantity above the analytical cut-off based on the C_q_, namely $\frac{35-Cq}{log2\left(10\right)}$. A weighted population AF for each pathogen was then calculated for any stratum of *j* cases as $AF=1–\frac{\sum_{1}^{j}\frac{wt_{i}}{OR_{i}}}{\sum_{1}^{j}wt_{i}}$, where *wt_i_* is the episode-specific inverse probability weight, and *OR_i_* is the episode-specific and quantity-specific OR derived from the regression model.^21^ We propagated uncertainty from the AF estimates using a Monte Carlo approach, which simulated 1000 new OR estimates from a normal distribution with the mean derived from the model coefficients and variance–covariance from the covariance matrix.^1^ To optimise the alignment between GPDS sites and the sites in which attribution modelling had been previously performed, these 1000 estimates were obtained using site-specific model coefficients from GEMS and MAL-ED with a draw distribution determined by weights that minimised the root mean square error distance between the pathogen density distribution in cases at each GPDS site and the proportionally weighted aggregate distribution across the GEMS and MAL-ED sites. In a separate sensitivity analysis, 1000 AF estimates were obtained evenly from the 15 site-specific model coefficients. Point estimates and 95% CIs were then calculated as the median and 2.5th and 97.5th quantiles, respectively.

To estimate diarrhoea incidence, we used national-level estimates of the number of diarrhoea hospitalisations, deaths and under 5 populations in 2017 from the Global Burden of Disease (GBD) 2019 study. Estimates of the incidence of diarrhoea and diarrhoeal deaths as well as the under 5 population for 2017 were obtained as previously described.^22 23^ To estimate diarrhoea hospitalisations, we used the incidence of hospital admissions from International Classification of Diseases codes due to diarrhoea from 67 sources and 42 countries and found the ratio of hospital admissions to all diarrhoea episodes. This proportion represented the proportion of diarrhoea that required hospitalisation. We modelled this value using DisMod-MR 2.1, a hierarchical Bayesian meta-regression model^1^ with the healthcare access and quality index as a predictor.^24^ We then estimated the proportion of diarrhoea episodes in children under 5 that were hospitalised for each GPDS country and multiplied these by diarrhoea incidence to obtain the incidence of hospitalised diarrhoea. To estimate variance for all of these estimates, 1000 draw values were obtained from the posterior distribution and propagated through the analyses.

To obtain AF or attributable incidence (AI) estimates across multiple countries, country-level estimates were aggregated. First, for countries with more than one surveillance site, AFs were combined as the mean of each of the 1000 estimates, save for estimates stratified by vaccination status, where AF estimates from India were included independently from each site due to state-level rotavirus vaccine introduction. Then, for each country and pathogen, we calculated the number of pathogen-attributable hospitalisations as the product of one randomly sampled draw of the country-level AF and one randomly sampled draw of the number of diarrhoea hospitalisations; this was repeated 10 000 times. Each estimate was summed across all included countries to obtain the total number of attributable episodes for each pathogen and was then either divided by the under 5 population denominator to obtain AI estimates or divided by the total number of diarrhoea hospitalisations to obtain AF estimates. An analogous analysis was conducted to obtain attributable episodes and incidence estimates for diarrhoeal deaths.

Lastly, to extrapolate from GPDS countries to estimate global and regional all-cause and aetiology-specific diarrhoeal deaths, we used the GBD country-level estimates to obtain 1000 estimates of the proportion of diarrhoeal deaths in each WHO region that were from GPDS countries. For the African and South-East Asian Regions, we further stratified these estimates by countries that had and had not introduced rotavirus vaccine, and this stratification was performed at the state level for India due to state-level rotavirus vaccine introduction. We then divided the total number of all-cause and pathogen-attributable deaths in GPDS countries in each regional stratum by this proportion. Because no countries from the Eastern Mediterranean region (EMR) were included in GPDS, regional AF estimates for the African region were applied to the EMR countries located in Africa, and those for the South-East Asian region were applied to the EMR countries located in Asia. These regional estimates were then summed to obtain global estimates. For all analyses, point estimates and 95% CIs were derived from the median and 2.5th and 97.5th quantiles, respectively, of the estimate distributions. All analyses were conducted in R V.4.0.2.

### Patient and public involvement

Patients and the public were not involved in the surveillance.

### Reflexivity statement

To promote equitable authorship of research publications from international partnerships, we have included a reflexivity statement as a online supplemental appendix.

## Results

**Figure 1:**
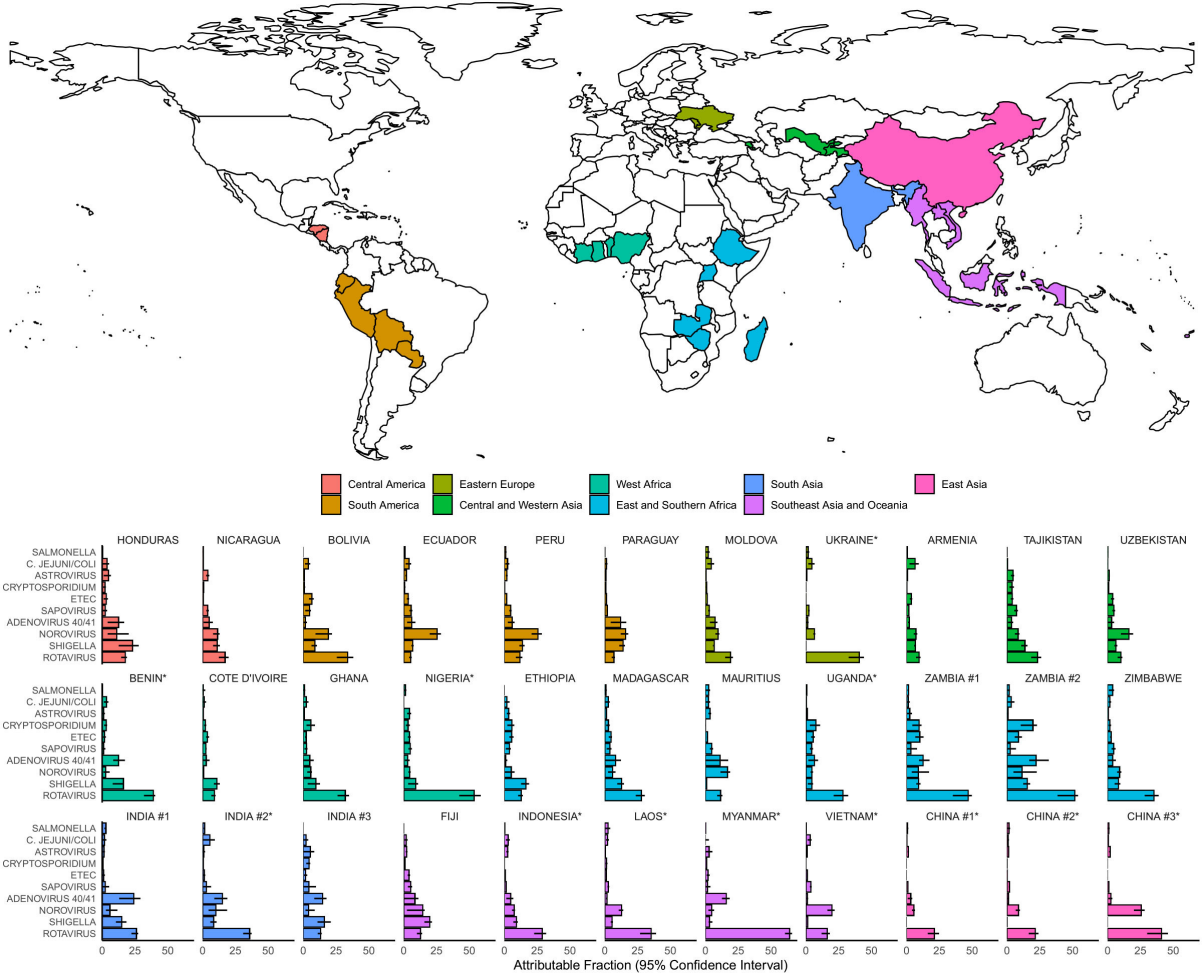
Pathogen-specific attributable fractions of hospitalised diarrhoea in children less than 5 years of age in 2017–2018 for the 33 surveillance sites from 28 countries in Global Pediatric Diarrhea Surveillance. The map indicates the country locations as well as their geographic groupings and the bar plots are coloured according to those groupings. Attributable fractions are expressed as a percent. *Rotavirus vaccine not introduced by 2017. ETEC, enterotoxigenic *E*. *coli*.

From January 2017 to December 2018, 29 502 children under 5 years of age hospitalised with diarrhoea were enrolled from 33 surveillance sites in 28 countries (online supplemental table 1 and figure 1). Using GBD incidence and population estimates, these 28 countries included 51.4% (299 119/582 295) of all estimated diarrhoeal deaths and 49.9% (337 863 737/677 362 399) of the estimated global under 5 population. Most enrolled children were less than 2 years of age (22716, 77.0%)(table 1). Most cases were accompanied by vomiting and dehydration, and almost all patients received some form of rehydration therapy in the hospital. Of 20 471 cases for which dehydration was present and the severity was estimated, 6681 (32.6%) had severe dehydration. Of 26 728 (90.6%) cases that could be classified by both duration and presence of blood, 24 616 (92.1%) presented with acute watery diarrhoea, 1728 (6.5%) with acute bloody diarrhoea and 384 (1.4%) with watery or bloody persistent diarrhoea.

**Table 1:** Demographic and clinical characteristics of patients less than 5 years of age hospitalised with diarrhoea enrolled in Global Pediatric Diarrhea Surveillance, 2017–2018

Demographic and clinical characteristics	All enrolled cases	qPCR-tested cases (unweighted)	qPCR-tested cases (weighted)*
N	29 502	5465	29 502
Male sex	17 171 (58.2)	3226 (59.0)	17 217 (58.4)
Female sex	12 331 (41.8)	2239 (41.0)	12 285 (41.6)
Age (months)			
0–11	12 828 (43.5)	2433 (44.5)	12 806 (43.4)
12–23	9888 (33.5)	1764 (32.3)	9785 (33.2)
24–59	6786 (23.0)	1268 (23.2)	6911 (23.4)
Calendar quarter			
January–March	6905 (23.4)	1161 (21.2)	6905 (23.4)
April–June	7262 (24.6)	1493 (27.3)	7262 (24.6)
July–September	8122 (27.5)	1384 (25.3)	8122 (27.5)
October–December	7213 (24.4)	1427 (26.1)	7213 (24.4)
WHO Region			
African	7880 (26.7)	1658 (30.3)	7880 (26.7)
Americas	5414 (18.4)	1012 (18.5)	5414 (18.4)
European	9164 (31.1)	1000 (18.3)	9164 (31.1)
South-East Asian	3104 (10.5)	839 (15.4)	3104 (10.5)
Western Pacific	3940 (13.4)	956 (17.5)	3940 (13.4)
Acute diarrhoea (<14 days)	29 055 (98.5)	5296 (98.5)	29 097 (98.6)
Bloody diarrhoea	1777 (6.6)	277 (5.4)	1614 (5.7)
Diarrhoeal duration (days)	2 (1–4)	2 (1–4)	2 (1–4)
Vomiting	20 074 (69.6)	3606 (68.0)	20 176 (69.7)
Dehydration	20 478 (75.8)	3412 (74.0)	20 227 (74.9)
Rehydration therapy given	26 288 (98.7)	4818 (98.1)	26 142 (99.2)
In-hospital deaths	125 (0.5)	35 (0.7)	98 (0.4)

Dichotomous estimates are shown as n (%), and continuous estimates are shown as median (IQR).

*Weighted Ns are rounded to the nearest integer.

Of these 26 728 cases, 25 688 (96.1%) had a stool sample collected and were eligible for random selection, of which 5465 (21.3%) were selected and tested by qPCR (online supplemental table 1). The demographic and clinical characteristics of the qPCR-tested cases were similar to those characteristics for all cases enrolled in GPDS after application of inverse probability of selection weights (table 1). Rotavirus was detected in 6246 of 25 983 children (24.0%) tested by EIA and in 1156 of 5041 children selected for qPCR testing (22.9%), while the weighted proportion in children selected for qPCR testing was 23.9%. Among all cases tested, the most prevalent pathogen detected, regardless of quantity, was rotavirus (weighted prevalence 9896, 33.5%), followed by adenovirus 40/41 (5667, 19.2%), norovirus (5324, 18.0%), *Shigella* (4224, 14.3%) and sapovirus (2938, 10.0%), with 12 pathogens present in at least 1% of stool samples (online supplemental figure 2).

The top aetiologies of diarrhoea were consistent across this wide range of low-income and middle-income countries (figure 1 and online supplemental table 3). The leading aetiology of diarrhoea was rotavirus at 22 of 33 sites (66.7%), norovirus at 6 (18.2%) and *Shigella* at 5 (15.2%). Four pathogens had an AF of more than 5% at the majority of sites (rotavirus at all 33 sites, norovirus at 27 (81.8%), *Shigella* at 25 (75.8%) and adenovirus 40/41 at 18 (54.5%)). Enterotoxigenic *Escherichia coli* (ETEC) and *Cryptosporidium* had AFs of more than 5% at five sites, and for both pathogens, four of these sites were in East and Southern Africa.

**Figure 2:**
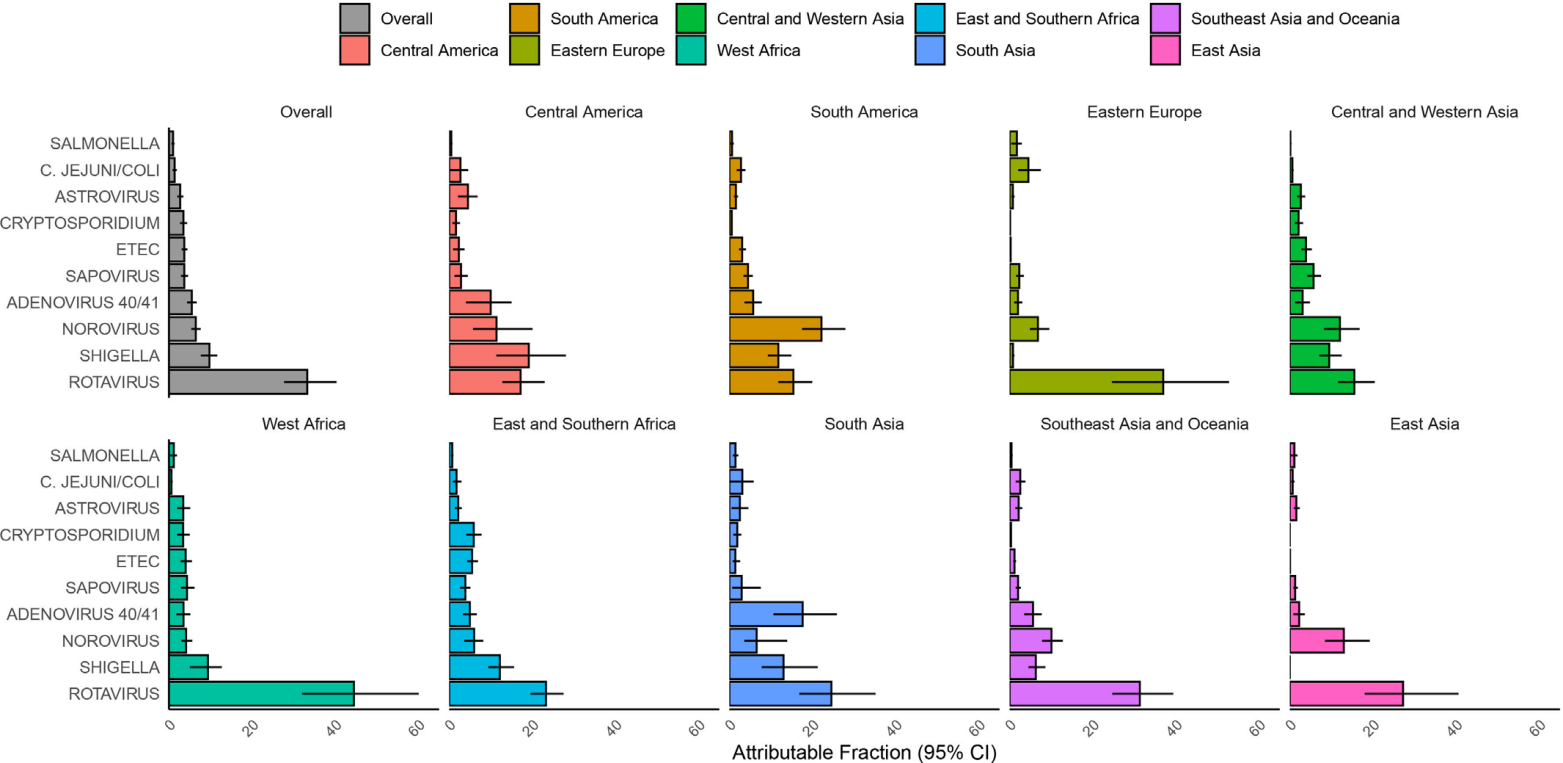
Pathogen-specific attributable fractions of hospitalised diarrhoea in children less than 5 years of age in 2017–2018 in Global Pediatric Diarrhea Surveillance both overall and by geographic region. Within each grouping and pathogen to attributable fractions were weighted by the site-level attributable incidence of hospitalised diarrhoea. Attributable fractions are expressed as a per cent. ETEC, enterotoxigenic *E*. *coli*.

In this network, 72.6% of hospitalised diarrhoea was attributable to one of the pathogens included in this analysis, ranging from 47.3% in East Asia to 79.2% in West Africa and South Asia (figure 2 and online supplemental table 4). Overall, the leading aetiologies of hospitalised diarrhoea were rotavirus (AF 33.3%; 95% CI 27.7 to 40.3), *Shigella* (9.7%; 95% CI 7.7 to 11.6), norovirus (6.5%; 95% CI 5.4 to 7.6) and adenovirus 40/41 (5.5%; 95% CI 4.4 to 6.7). Rotavirus was the leading aetiology of diarrhoea in all regions, with the exception of the Americas, where all countries included in GPDS had introduced rotavirus vaccine into their national immunisation programme by 2010. In Central America, *Shigella* was the leading aetiology (AF 19.2%; 95% CI 11.4 to 28.1), while in South America, norovirus was the leading aetiology (22.2%; 95% CI 17.5 to 27.9). Some pathogens were particularly important for certain geographic settings, including adenovirus 40/41 in South and Southeast Asia, *Vibrio cholerae* in India and ETEC and *Cryptosporidium* in Africa.

Except for rotavirus and norovirus in the Americas and the Western Pacific, aetiology estimates were stable between 2017 and 2018 (online supplemental figure 3). Rotavirus was the leading aetiology of hospitalised diarrhoea across all age groups (0–11 months: AF 32.5%; 95% CI 26.6 to 39.6; 12–23 months: 33.5%; 95% CI 28.2 to 39.8; 24–59 months: 29.7%; 95% CI 25.1 to 35.1), while *Shigella* increased substantially in older age groups (0–11 months: AF 5.1%; 95% CI 3.8 to 6.2; 12–23 months: 12.9%; 95% CI 9.9 to 15.7; 24–59 months: 17.9%; 95% CI 14.0 to 21.4) (online supplemental table 5 and figure 4). Rotavirus was the leading aetiology of acute, watery diarrhoea, while *Shigella* was the leading aetiology of both bloody and persistent diarrhoea (online supplemental figure 5). The aetiological distribution was similar between males and females (online supplemental figure 6).

**Figure 3:**
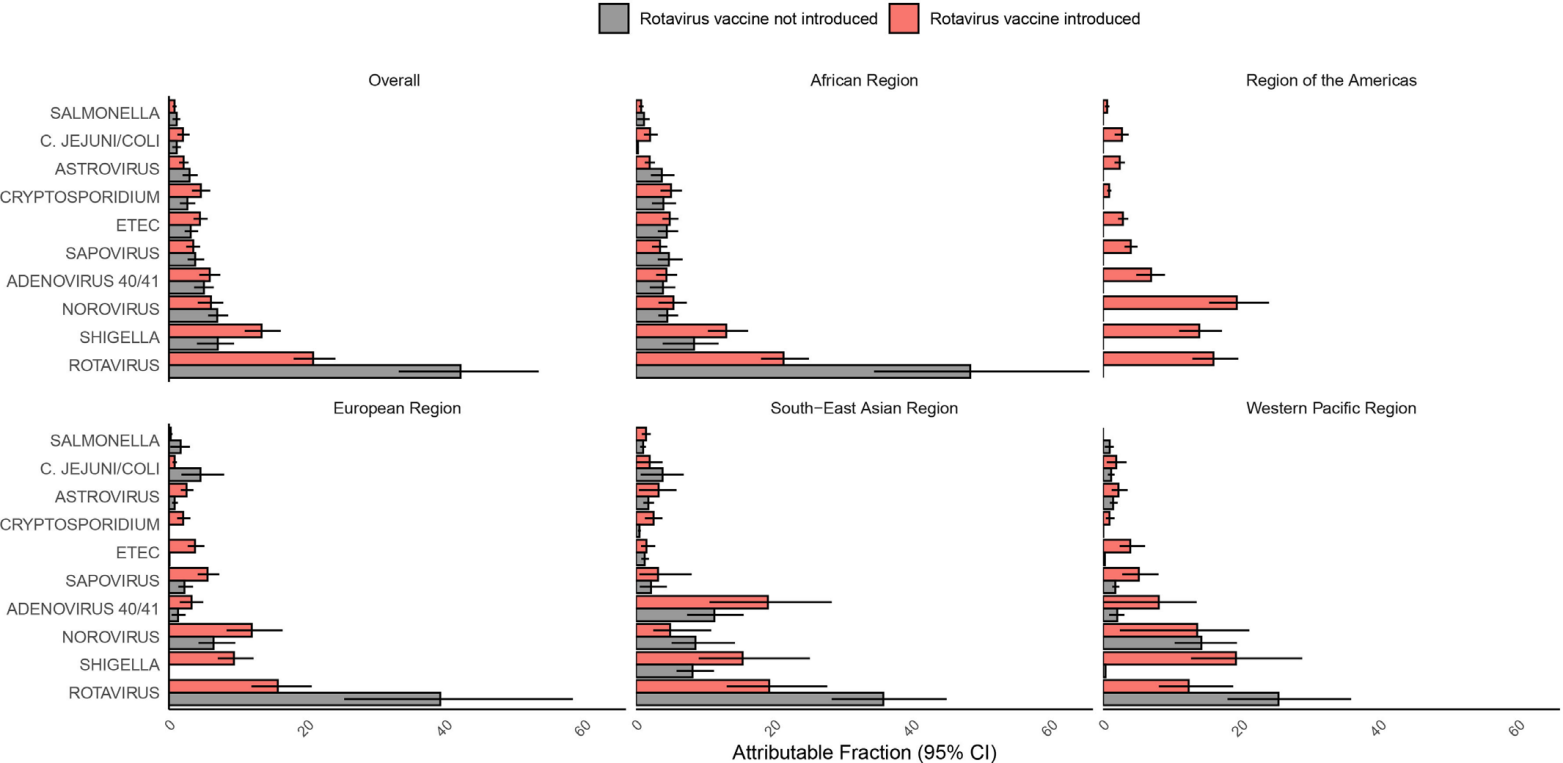
Pathogen-specific attributable fractions of hospitalised diarrhoea in children less than 5 years of age in 2017–2018 in Global Pediatric Diarrhea Surveillance both overall and by geographic region to by rotavirus vaccination introduction status as of 2017. For each grouping, attributable fractions were weighted by the site-level attributable incidence of hospitalised diarrhoea. Attributable fractions are expressed as a percent. ETEC, enterotoxigenic *E*. *coli*.

Rotavirus vaccines had been introduced at 21 (63.6%) of the 33 surveillance sites by the end of 2017. We compared the AF of rotavirus at sites where rotavirus vaccine had been introduced to those where it had not been introduced (figure 3 and online supplemental table 6). Overall, the rotavirus AF was slightly more than 50% lower in sites that had introduced rotavirus vaccine (AF 20.8%; 95% CI 18.0 to 24.1) compared with sites that had not (42.1%; 95% CI 33.2 to 53.4). Across the included WHO regions, the rotavirus AF was between 45% and 60% lower in sites that had introduced rotavirus vaccine. However, even in sites that had introduced rotavirus vaccine, rotavirus remained the leading aetiology of hospitalised diarrhoea both overall and in the African, European and South-East Asian regions. In the South-East Asian region, the burden of rotavirus (19.2%; 95% CI 13.1 to 27.6) and adenovirus 40/41 (19.0%; 10.6 to 28.3) was similar in countries that had introduced rotavirus vaccination.

Comparing the overall AF with the prevalence of each pathogen using Cq cut-offs of 35 (the analytical limit of detection) and 30 (a more conservative cut-off to exclude low-quantity pathogen detections), the same five pathogens had the highest burden using all three metrics, and the overall weighted prevalence using a Cq cut-off of 30 roughly approximated the overall AF (online supplemental figure 7). In comparison with AFs estimated using an even number of draws from each site-specific model (online supplemental figure 8), the overall AF estimate using an optimised draw distribution changed by more than 20% for only two of the top 10 pathogens: norovirus (from 4.9% to 6.5%; +31.2%) and *Campylobacter jejuni/coli* (from 1.1 to 1.4%; +24.3%).

Using GBD incidence estimates for the GPDS countries, the incidence of all-cause diarrhoea hospitalisations was 19.2 per 1000 child-years (95% CI 17.0 to 21.9) and ranged from 3.9 (95% CI 2.8 to 5.4) in Eastern Europe to 61.3 (95% CI 48.0 to 78.4) in West Africa and 60.2 (95% CI 50.4 to 72.2) in East and Southern Africa. Overall, rotavirus had the highest AI of hospitalised diarrhoea in children (AI 6.4; 95% CI 5.3 to 7.8), followed by *Shigella* (1.9; 95% CI 1.5 to 2.2), norovirus (1.2; 95% CI 1.0 to 1.5) and adenovirus 40/41 (1.1; 95% CI 0.8 to 1.3) (table 2). The incidence of all-cause diarrhoeal deaths was 88.5 per 100 000 child-years (95% CI 76.2 to 103.0) and ranged from 0.8 (95% CI 0.6 to 1.1) in Eastern Europe to 358.1 (95% Ci 280.0 to 459.2) in West Africa. Rotavirus had the highest incidence of fatal diarrhoea (AI 34.0 per 100 000 child-years; 95% CI 27.1 to 42.2), followed by *Shigella* (9.6; 95% CI 6.9 to 12.0), norovirus (6.3; 95% CI 4.7 to 7.8) and adenovirus 40/41 (4.9; 95% CI 3.9 to 6.4).

**Table 2:** Pathogen-specific attributable incidence with 95% CIs of diarrhoea hospitalisations and deaths in 2017–2018 both globally and by geographic grouping in children less than 5 years of age in Global Pediatric Diarrhea Surveillance countries

	All cause	Rotavirus	*Shigella*	Norovirus	Adenovirus 40/41	Sapovirus	ETEC	*Cryptosporidium*	Astrovirus	*C. jejuni/C. coli*	*Salmonella*
Hospitalisations*	19.2 (17.0 to 21.9)	6.4 (5.3 to 7.8)	1.9 (1.5 to 2.2)	1.2 (1.0 to 1.5)	1.1 (0.8 to 1.3)	0.7 (0.6 to 0.9)	0.7 (0.6 to 0.9)	0.7 (0.5 to 0.8)	0.5 (0.4 to 0.7)	0.3 (0.2 to 0.4)	0.2 (0.1 to 0.3)
Central America	17.2 (13.2 to 22.3)	3.0 (2.2 to 4.0)	3.3 (1.9 to 4.8)	2.0 (1.0 to 3.5)	1.7 (0.7 to 2.6)	0.5 (0.2 to 0.8)	0.4 (0.2 to 0.6)	0.3 (0.1 to 0.5)	0.8 (0.4 to 1.2)	0.5 (0.0 to 0.8)	0.1 (0.0 to 0.1)
South America	10.8 (8.9 to 13.1)	1.7 (1.3 to 2.2)	1.3 (1.0 to 1.6)	2.4 (1.9 to 3.0)	0.6 (0.4 to 0.8)	0.5 (0.4 to 0.6)	0.3 (0.2 to 0.4)	0.1 (0.0 to 0.1)	0.2 (0.1 to 0.2)	0.3 (0.2 to 0.4)	0.1 (0.0 to 0.1)
Eastern Europe	3.9 (2.8 to 5.4)	1.4 (1.0 to 2.1)	0.0 (0.0 to 0.0)	0.3 (0.2 to 0.4)	0.1 (0.0 to 0.1)	0.1 (0.1 to 0.1)	0.0 (0.0 to 0.0)	0.0 (0.0 to 0.0)	0.0 (0.0 to 0.0)	0.2 (0.1 to 0.3)	0.1 (0.0 to 0.1)
Central and Western Asia	4.4 (3.4 to 5.6)	0.7 (0.5 to 0.9)	0.4 (0.3 to 0.5)	0.5 (0.4 to 0.7)	0.1 (0.1 to 0.2)	0.3 (0.2 to 0.3)	0.2 (0.1 to 0.2)	0.1 (0.1 to 0.1)	0.1 (0.1 to 0.2)	0.0 (0.0 to 0.0)	0.0 (0.0 to 0.0)
West Africa	61.3 (48.0 to 78.4)	27.5 (19.7 to 36.9)	5.8 (3.1 to 7.8)	2.6 (1.8 to 3.4)	2.2 (1.1 to 3.2)	2.7 (1.8 to 3.8)	2.5 (1.7 to 3.4)	2.1 (1.2 to 3.1)	2.1 (1.2 to 3.2)	0.4 (0.2 to 0.6)	0.7 (0.2 to 1.2)
East and Southern Africa	60.2 (50.4 to 72.2)	14.1 (11.8 to 16.6)	7.4 (5.8 to 9.4)	3.6 (2.2 to 5.0)	3.0 (2.0 to 4.1)	2.4 (1.6 to 3.1)	3.3 (2.6 to 4.2)	3.6 (2.5 to 4.7)	1.3 (0.8 to 1.8)	1.1 (0.5 to 1.8)	0.4 (0.3 to 0.6)
South Asia	4.9 (3.4 to 7.1)	1.2 (0.8 to 1.8)	0.7 (0.4 to 1.1)	0.3 (0.2 to 0.7)	0.9 (0.5 to 1.3)	0.2 (0.0 to 0.4)	0.1 (0.0 to 0.1)	0.1 (0.0 to 0.1)	0.1 (0.0 to 0.2)	0.2 (0.0 to 0.3)	0.1 (0.0 to 0.1)
Southeast Asia and Oceania	14.3 (11.5 to 17.8)	4.5 (3.6 to 5.7)	0.9 (0.7 to 1.2)	1.5 (1.1 to 1.8)	0.8 (0.5 to 1.1)	0.3 (0.2 to 0.4)	0.2 (0.1 to 0.2)	0.0 (0.0 to 0.1)	0.3 (0.2 to 0.4)	0.4 (0.2 to 0.5)	0.1 (0.0 to 0.1)
East Asia	6.2 (4.2 to 9.1)	1.7 (1.1 to 2.5)	0.0 (0.0 to 0.0)	0.8 (0.5 to 1.2)	0.1 (0.0 to 0.2)	0.1 (0.1 to 0.1)	0.0 (0.0 to 0.0)	0.0 (0.0 to 0.0)	0.1 (0.1 to 0.2)	0.0 (0.0 to 0.1)	0.1 (0.0 to 0.1)
Deaths†	88.5 (76.2 to 103.0)	34.0 (27.1 to 42.2)	9.6 (6.9 to 12.0)	6.3 (4.7 to 7.8)	4.9 (3.9 to 6.4)	3.6 (2.6 to 4.9)	3.3 (2.6 to 4.1)	3.0 (2.0 to 3.9)	2.9 (2.0 to 3.8)	1.2 (0.4 to 1.8)	1.0 (0.5 to 1.4)
Central America	33.0 (22.2 to 47.7)	5.7 (3.7 to 8.5)	6.9 (3.6 to 11.5)	3.7 (1.3 to 6.0)	3.9 (1.6 to 7.8)	0.9 (0.3 to 1.6)	0.9 (0.3 to 1.6)	1.5 (0.6 to 2.6)	0.6 (0.3 to 1.1)	1.0 (0.0 to 1.9)	0.2 (0.1 to 0.3)
South America	11.6 (7.6 to 17.1)	2.1 (1.2 to 3.7)	1.3 (0.8 to 1.9)	0.6 (0.3 to 0.9)	2.6 (1.7 to 3.8)	0.5 (0.3 to 0.8)	0.4 (0.2 to 0.8)	0.2 (0.1 to 0.3)	0.1 (0.0 to 0.1)	0.4 (0.2 to 0.6)	0.1 (0.0 to 0.1)
Eastern Europe	0.8 (0.6 to 1.1)	0.3 (0.2 to 0.4)	0.0 (0.0 to 0.0)	0.0 (0.0 to 0.0)	0.1 (0.0 to 0.1)	0.0 (0.0 to 0.0)	0.0 (0.0 to 0.0)	0.0 (0.0 to 0.0)	0.0 (0.0 to 0.0)	0.0 (0.0 to 0.1)	0.0 (0.0 to 0.0)
Central and Western Asia	16.8 (11.0 to 25.9)	3.5 (2.1 to 5.7)	2.1 (1.3 to 3.4)	0.6 (0.2 to 1.0)	1.6 (1.0 to 2.4)	1.1 (0.7 to 1.8)	0.7 (0.4 to 1.1)	0.7 (0.4 to 1.1)	0.5 (0.2 to 1.0)	0.1 (0.0 to 0.1)	0.0 (0.0 to 0.0)
West Africa	358.1 (280.0 to 459.2)	182.0 (131.7 to 241.6)	33.2 (14.3 to 45.7)	12.1 (4.8 to 18.5)	16.0 (11.5 to 21.4)	17.3 (11.4 to 24.1)	15.4 (10.4 to 21.0)	14.7 (8.3 to 21.3)	12.1 (5.8 to 18.4)	1.1 (0.6 to 1.7)	4.5 (0.6 to 7.6)
East and Southern Africa	161.2 (128.3 to 210.4)	34.8 (27.6 to 43.8)	21.9 (16.3 to 30.4)	7.7 (4.5 to 10.9)	9.4 (4.9 to 13.9)	6.1 (3.7 to 8.7)	9.1 (6.8 to 12.4)	3.9 (2.4 to 6.0)	9.4 (6.1 to 13.3)	3.2 (1.4 to 5.6)	0.8 (0.4 to 1.2)
South Asia	52.8 (39.5 to 69.0)	13.1 (9.7 to 17.5)	7.0 (4.4 to 10.7)	9.5 (5.9 to 12.8)	3.5 (2.0 to 7.3)	1.6 (0.4 to 3.9)	0.8 (0.4 to 1.3)	1.4 (0.3 to 2.3)	1.0 (0.5 to 1.4)	1.7 (0.0 to 3.0)	0.8 (0.5 to 1.1)
Southeast Asia and Oceania	38.8 (30.3 to 49.0)	13.3 (10.1 to 17.3)	3.1 (2.2 to 4.2)	2.5 (1.4 to 3.6)	2.9 (2.1 to 3.7)	0.6 (0.4 to 0.8)	0.4 (0.3 to 0.6)	1.0 (0.6 to 1.4)	0.1 (0.1 to 0.2)	1.0 (0.4 to 1.6)	0.2 (0.1 to 0.4)
East Asia	2.3 (1.9 to 2.8)	0.6 (0.5 to 0.8)	0.0 (0.0 to 0.0)	0.1 (0.0 to 0.1)	0.3 (0.2 to 0.4)	0.0 (0.0 to 0.0)	0.0 (0.0 to 0.0)	0.0 (0.0 to 0.0)	0.0 (0.0 to 0.0)	0.0 (0.0 to 0.0)	0.0 (0.0 to 0.0)

*Per 1000 child-years.

†Per 100 000 child-years.

ETEC, enterotoxigenic *E. coli*.

Using the aetiology distribution as a proxy for the aetiology of diarrhoeal deaths, we then extrapolated to estimate annual diarrhoeal deaths for WHO regions and globally (table 3). The top causes of diarrhoeal deaths were rotavirus (208 009 annual deaths; 95% CI 169 561 to 2 59 216), *Shigella* (62 853; 95% CI 48 656 to 78805), adenovirus 40/41 (36 922; 95% CI 28 469 to 46 672) and norovirus (35 914; 95% CI 27 258 to 46 516). Sapovirus, ETEC, *Cryptosporidium*, astrovirus, *Campylobacter jejuni/coli* and *Salmonella* were also estimated to be the cause of more than 5000 deaths per year.

**Table 3:** Estimated all-cause and pathogen-attributable diarrhoeal deaths in 2017–2018 with 95% CIs both globally and by WHO region in children less than 5 years of age

	Global	African region	Region of the Americas	Eastern Mediterranean region*	European region	South-East Asian region	Western Pacific region
All cause	582 295 (493 241 to 683 788)	396 459 (321 310 to 482 064)	10 483 (7385 to 14 682)	79 661 (56 853 to 108 689)	1623 (1069 to 2597)	84 565 (70 943 to 101 038)	8175 (6057 to 10 868)
Rotavirus	208 009 (169 561 to 259 216)	148 931 (115 068 to 191 171)	1857 (1221 to 2898)	28 343 (20 445 to 39 430)	342 (207 to 560)	25 829 (20 780 to 31 466)	2283 (1590 to 3307)
*Shigella*	62 853 (48 656 to 78 805)	43 947 (30 852 to 57 086)	1570 (995 to 2376)	7837 (5221 to 11 774)	193 (116 to 319)	9164 (6608 to 11 997)	106 (54 to 211)
Adenovirus 40/41	36 922 (28 469 to 46 672)	15 117 (9339 to 20 597)	765 (439 to 1166)	8182 (5333 to 12 275)	54 (17 to 97)	12 701 (9130 to 16 202)	175 (100 to 250)
Norovirus	35 914 (27 258 to 46 516)	19 562 (13 936 to 26 002)	1843 (1201 to 2883)	5881 (3267 to 9851)	156 (96 to 243)	6960 (3958 to 11 553)	1094 (816 to 1475)
Sapovirus	22 704 (16 452 to 29 354)	17 060 (12 249 to 22 275)	396 (245 to 605)	2539 (1176 to 4507)	108 (65 to 185)	2302 (578 to 4550)	143 (103 to 208)
ETEC	22 530 (17 762 to 28 869)	18 879 (14 817 to 24 304)	338 (205 to 559)	1988 (1224 to 2971)	63 (37 to 109)	1158 (726 to 1700)	28 (17 to 52)
*Cryptosporidium*	19 905 (14 364 to 26 984)	17 121 (11 950 to 23 540)	116 (62 to 194)	1553 (949 to 2527)	51 (22 to 95)	984 (664 to 1278)	22 (8 to 55)
Astrovirus	17 213 (12 095 to 22 573)	13 208 (8547 to 18 064)	289 (164 to 460)	1832 (1077 to 2773)	63 (32 to 110)	1670 (862 to 2477)	110 (80 to 151)
*C. jejuni/C. coli*	9741 (4023 to 15 478)	4130 (2144 to 6778)	321 (150 to 503)	2263 (372 to 4468)	9 (5 to 16)	3032 (291 to 5388)	92 (53 to 153)
*Salmonella*	6021 (3391 to 8442)	3688 (1188 to 5794)	55 (29 to 97)	965 (614 to 1404)	1 (0 to 3)	1160 (788 to 1450)	104 (44 to 175)

*Aetiology-specific diarrhoeal deaths for countries in the Eastern Mediterranean region were estimated using attributable fractions from the African region for countries that are physically in Africa and from the South-East Asian region for countries that are physically in Asia.

ETEC, enterotoxigenic *E. coli*.

## Discussion

By incorporating quantitative molecular diagnostics and pathogen attribution modelling into a global network with standardised surveillance, sample collection and testing, this study provides direct estimates of the aetiology of diarrhoea requiring hospitalisation in children under 5 years of age in LMICs in 2017–2018. Despite the substantial and consistent impact of rotavirus vaccine introduction across geographies, rotavirus remained the leading cause of diarrhoea hospitalisations. We estimated that rotavirus was still responsible for one in three diarrhoeal hospitalisations in these populations. By focusing on this most severe subset of diarrhoea, we additionally identified *Shigella*, adenovirus 40/41 and norovirus as pathogens with a high burden of disease and global relevance. While these four pathogens had a consistently high attributable burden of diarrhoea, a wide range of additional pathogens accounted for the remaining pathogen-attributable hospitalisations with significant geographic variation. The estimates were robust to several assumptions around the diagnostic and analytical approaches and due to the inclusion of a breadth of LMICs representing about half of the total global disease burden, the generalisability of the aggregated results to all LMICs should be high.

The impact of rotavirus vaccine introduction seen in this analysis was consistent with the 40% decline in rotavirus prevalence after vaccine introduction in the broader GRSN.^2^ However, rotavirus remained the leading cause of severe paediatric diarrhoea in LMICs outside of the Americas. The rotavirus AF remained high in some sites that had introduced rotavirus vaccine, a finding that might be explained by a number of factors including poor vaccine coverage, a high baseline burden of disease or a low prevalence of other pathogens. Individual vaccine status for patients enrolled was not available, nor was vaccine coverage in individual site catchment areas, thus we could not directly evaluate the impact of vaccine coverage. These findings support the need to continue to extend rotavirus vaccine introduction and coverage. WHO recommends that every country include rotavirus vaccine in their infant immunisation programmes,^25^ but fewer than half of infants born in 2018 globally were immunised against rotavirus.^26^ Efforts to improve the performance of rotavirus vaccines, which currently have 50–60% efficacy in low-income settings, are also critically important.^27^

This network, which added molecular diagnostic testing to a subset of sites from a broader existing surveillance system for rotavirus, provides a broader context for the relatively importance of additional enteric vaccines as well as a platform for evaluating vaccine impact after introduction.^4^ While vaccines against cholera are available, WHO recommends use of cholera vaccines only for specific endemic settings and during outbreaks,^28^ the burden of which cannot be adequately evaluated via sentinel surveillance. Vaccines against *Shigella* and norovirus are in development, and our findings support their broad potential impact on childhood morbidity and mortality in these settings.^29 30^ Finally, these data suggest the need for options to control and reduce the burden of enteric adenoviruses, which were also found to be common across many regions.^31^

Using diarrhoea hospitalisations as a proxy for diarrhoea mortality, our estimates of pathogen-specific diarrhoeal deaths showed some similarities as well as some important differences compared with the most recent modelled estimates.^1^ Similar to these estimates, we found rotavirus and *Shigella* to be the leading causes of diarrhoeal deaths, but we identified a substantially higher burden of norovirus and a lower burden of *Cryptosporidium*, *Campylobacter* and *Salmonella*. Norovirus is widely estimated to be the leading aetiology of hospitalised paediatric diarrhoea in high-income countries, but prior estimates of norovirus burden have been lower from LMICs.^32^ The low burden of *Cryptosporidium* was unexpected and may reflect that our surveillance sites miss areas of significant endemicity for this pathogen or that prior estimates extrapolated inappropriately from such areas. In our earlier pilot study,^17^ we found that *Cryptosporidium* was the second most common aetiology of diarrhoea in African countries, in contrast to being the fifth most common in this study. Seven of the 12 African countries included in the pilot were not included in this study because they did not meet the site inclusion criteria, and this study included an additional five countries from this region. In addition, the pilot study was from 2013 to 2014, 3–4 years prior to this study, and HIV infections in children have declined in Africa during this period,^33^ which could also contribute to the relatively low burden of *Cryptosporidium* found in the present study. *Campylobacter* and *Salmonella* may be relatively less likely to cause severe acute dehydration,^10^ and it is possible that the extrapolation of pathogen prevalence data from studies of non-hospitalised diarrhoea, for example, in studies such as GBD, may lead to higher estimates for these pathogens. This study makes a substantial contribution of data for reconciling these differences and improving model-based estimates. These findings are also mostly consistent with two multisite paediatric diarrhoea aetiology studies, GEMS and MAL-ED, which were performed across 15 sites in Asia, Africa and South America from 2007 to 2012 using the same diagnostic platform and provided the data for the attribution modelling used in this analysis.^8–10^ In those studies, the burden of norovirus was variable, and ETEC and *Cryptosporidium* were more consistently identified as important pathogens than in the present study. Approximately 20% of subjects in GEMS were hospitalised, while MAL-ED was a community-based cohort study in which hospitalisation for diarrhoea was rare.

We were able to identify an aetiology in approximately three-quarters of cases, which was within the bounds of the proportion of cases without an identified aetiology in multisite studies of community (MAL-ED: 65%)^9^ and moderate-to-severe diarrhoea (GEMS: 89%)^8^ using the same diagnostic platform. Relatively high-income sites included in GPDS, including in East Asia and Eastern Europe, where the pretest probability for infectious diarrhoea is likely lower, had less cumulative attribution to the included pathogens. This study did not enrol concurrent controls but rather used attribution models developed in these previous studies.^8 9^ These studies did not include countries from Europe and few from the Americas and used different case definitions than GPDS. Because these regions are expected to have less subclinical carriage of enteropathogens, the attribution models may have been overly conservative in assigning aetiology in these sites. The use of optimised draw distributions from the attribution models based on pathogen density in cases was designed to close this gap in settings with lower pathogen carriage. This approach increased attribution to norovirus and *Campylobacter* in particular, pathogens with particularly a high subclinical prevalence in highly endemic settings. Additionally, the overall weighted prevalence using a conservative Cq cut-off approximated the weighted AF estimate for the top pathogens, suggesting that our estimate of the aetiology hierarchy was robust to these assumptions around pathogen attribution.

Several other limitations are important to note. First, GPDS sentinel surveillance hospitals are predominantly but not exclusively referral hospitals in urban centres and thus may not be representative of all hospitalised diarrhoea in each country. Second, these sites were initially chosen for inclusion in the Global Rotavirus Surveillance Network. While we have expanded the case definition to all hospitalised diarrhoea, there may be some bias towards sites with high rotavirus incidence due to the initial site selection. Additionally, hospitalisation may not be a consistent indicator of disease severity across diverse settings. Third, while underlying malnutrition is a major risk factor for diarrhoea hospitalisation and death,^34 35^ nutritional status indicators were not collected from enrolled children, and thus we could not explore the impact of nutritional status on aetiology. Third, no countries from the Eastern Mediterranean Region were included and thus death estimates for this region required an assumption that diarrhoea aetiology from geographically proximate sites was representative. Fourth, because these sites do not have defined catchment areas, our extrapolation to incidence of diarrhoea hospitalisations and deaths relied on GBD model estimates. Finally, as healthcare access continues to improve, it is possible that the proxy of diarrhoea requiring hospitalisation will become less applicable for understanding the aetiology of fatal diarrhoea.

## Conclusion

By leveraging existing sentinel surveillance in a globally representative range of LMICs and applying the best-available diagnostic and analytical methods, we provide a novel picture of the aetiology of hospitalised diarrhoea in children under 5 and substantially increase the available data for estimating aetiology-specific diarrhoeal deaths. These data identify a significant residual burden of rotavirus diarrhoea and associated deaths and identify *Shigella*, norovirus and adenovirus 40/41 as additional pathogens of global importance.

## Data Availability

Data are available on reasonable request. The deidentified participant-level data used in these analyses will be made available on request to qualified researchers after publication of this manuscript, after approval of a proposal submitted to the corresponding author and signing of a data access agreement.
