# Supplementary material for: Aetiology and incidence of diarrhoea requiring hospitalisation in children under 5 years of age in 28 low-income and middle-income countries: findings from the Global Pediatric Diarrhea Surveillance network
Source: BMJ Glob Health. 2022 Sep 5;7(9):e009548. doi: 10.1136/bmjgh-2022-009548 (PMC9445824; doi:10.1136/bmjgh-2022-009548)
Supplement: Supplementary data [file bmjgh-2022-009548supp001.pdf]

Supplementary material

Supplementary Table 1. Sentinel hospital surveillance sites participating in Global Pediatric Diarrhea Surveillance, 2017-2018 2

Supplementary Table 2. Testing for established aetiologies of diarrhoea by qPCR on the TaqMan Array Card and corresponding gene target........ 3

Supplementary Table 3. Pathogen-specific attributable fractions with 95% confidence intervals of diarrhoea hospitalizations by Global Pediatric Diarrhea Surveillance site in children less than 5 years of age, 2017-2018 (Figure 1). 4

Supplementary Table 4. Pathogen-specific attributable fractions with 95% confidence intervals of diarrhoea hospitalizations both overall and by geographic grouping in children less than 5 years of age in countries participating in Global Pediatric Diarrhea Surveillance, 2017-2018 (Figure 2). 5

Supplementary Table 5. Pathogen-specific attributable fractions with 95% confidence intervals of diarrhoea hospitalizations by age in children less than 5 years of age in countries participating in Global Pediatric Diarrhea Surveillance, 2017-2018 (Supplementary Figure 4)........ 6

Supplementary Table 6. Pathogen-specific attributable fractions with 95% confidence intervals of diarrhoea hospitalizations both overall and by World Health Organization region, stratified by introduction of rotavirus vaccine by 2017, in children less than 5 years of age in countries participating in Global Pediatric Diarrhea Surveillance, 2017-2018 (Figure 3). 7

Supplementary Figure 1. Diarrhoea surveillance and qPCR testing by 3-month calendar quarters at each Global Pediatric Diarrhea Surveillance site, 2017-2018........ 8

Supplementary Figure 2. Weighted prevalence by qPCR at quantification cycle cut-offs of 35 and 30 of diarrhoeal pathogens by Global Pediatric Diarrhea Surveillance site, 2017-2018. 9

Supplementary Figure 3. Pathogen-specific attributable fractions by World Health Organization region and year. 10

Supplementary Figure 4. Pathogen-specific attributable fractions by age. 11

Supplementary Figure 5. Pathogen-specific attributable fractions by diarrhoeal syndrome........12

Supplementary Figure 6. Pathogen-specific attributable fractions by sex. 13

Supplementary Figure 7. Overall weighted prevalence of diarrhoeal pathogens in cases by qPCR at quantification cycle cut-offs of 35 and 30 and comparison to weighted attributable fractions in Global Pediatric Diarrhea Surveillance, 2017-2018. Cq = quantification cycle........14

Supplementary Figure 8. Pathogen-specific attributable fractions estimated using even vs. optimized draws from the GEMS and MAL-ED models. 15

References........16

Cohen AL, *et al. BMJ Global Health* 2022; 7:e009548. doi: 10.1136/bmjgh-2022-009548

Supplementary Table 1. Sentinel hospital surveillance sites participating in Global Pediatric Diarrhea Surveillance, 2017-2018

WHO Region	Geographic Grouping	Country	Surveillance Site	Regional Reference Laboratory*	Date range included	Cases enrolled	qPCR-tested cases	Rotavirus vaccine introduction
African (AFR)	West Africa	Benin	Hôpital de Zone Suru Lere, Cotonou	Ghana	April 2017 – December 2018	305	157 (51·5)	Not introduced by end of 2018
	West Africa	Côte d'Ivoire	Yopougon University Hospital, Centre Hospitalier Universitaire de Cocody, Hôpital General Port Bouet, Abidjan	Ghana	April 2017 – December 2018	349	136 (39·0)	2017
	East and Southern Africa	Ethiopia	Tikur Anbessa (Black Lion) Hospital and Yekatit 12 Hospital, Addis Ababa	South Africa	April 2017 – December 2018	259	168 (64·9)	2013
	West Africa	Ghana	Korle-Bu Teaching Hospital, Accra	Ghana	April 2017 – December 2018	435	143 (32·9)	2012
	East and Southern Africa	Madagascar	Tsaralalana Mother and Child Hospital, Antananarivo	South Africa	April 2017 – December 2018	526	146 (27·8)	2014
	East and Southern Africa	Mauritius	Jwaharlal Nehru Hospital, Rose Belle, and Dr. AG Jeetoo Hospital, Port Louis	South Africa	January 2017 – December 2018	432	155 (35·9)	2015
	West Africa	Nigeria	Institute of Child Health: University of Nigeria Teaching Hospital, Enugu	Ghana	April 2017 – December 2018	626	144 (23·0)	Not introduced by end of 2018
	East and Southern Africa	Uganda	Mulago National Referral Hospital, Kampala	South Africa	April 2017 – December 2018	1109	172 (15·5)	2018
	East and Southern Africa	Zambia #1	University Teaching Hospital, Lusaka	South Africa	April 2017 – December 2018	818	143 (17·5)	2013
		Zambia #2	Arthur Davison Children's Hospital, Ndola	South Africa	April 2017 – December 2018	540	146 (27·0)	2013
	East and Southern Africa	Zimbabwe	Chitungwiza Central Hospital, Central Harare Hospital and Parirenyatwa Group of Hospitals, Harare	South Africa	April 2017 – December 2018	2481	148 (6·0)	2014
American (AMR)	South America	Bolivia	Centro Pediãtrico Albina Patiã'o, Cochabamba	Brazil	January 2018 – December 2018	260	115 (44·2)	2008
	South America	Ecuador	Hospital de Niã'os Baca Ortiz, Quito	Brazil	January 2017 – December 2018	475	178 (37·5)	2008
	Central America	Honduras	Hospital Escuela, Tegucigalpa	USA	January 2017 – December 2018	1137	193 (17·0)	2009
	Central America	Nicaragua	Hospital Infantil Manuel De Jesus Rivera, Managua	USA	January 2017 – December 2018	2707	209 (7·7)	2007
	South America	Peru	Hospital Goyeneche de Arequipa, Arequipa	Brazil	January 2017 – December 2018	543	175 (32·2)	2008
	South America	Paraguay	Hospital de Niños de Acosta Ñú "Reducto" San Lorenzo, San Lorenzo	USA	January 2017 – December 2018	292	142 (48·6)	2010
European (EUR)	Central and Western Asia	Armenia	Infectious Disease Hospital Nork, Yerevan	Belarus	January 2017 – December 2018	1092	200 (18·3)	2012
	Eastern Europe	Moldova	Children Infectious Clinical Hospital and Children Clinical Hospital No.1, Chisinau	Belarus	January 2017 – December 2018	2016	200 (9·9)	2012
	Central and Western Asia	Tajikistan	Children's Hospital of Infectious Diseases, Dushanbe	Belarus	January 2017 – December 2018	2791	200 (7·2)	2015
	Eastern Europe	Ukraine	Kiev City Children Clinical Hospital No.1, Kiev	Belarus	January 2017 – December 2018	1170	200 (17·1)	Not introduced by end of 2018
	Central and Western Asia	Uzbekistan	Infectious Diseases Hospital # 4, Tashkent	Belarus	January 2017 – December 2018	2095	200 (9·5)	2014
South-East Asian (SEAR)	South Asia	India #1	Christian Medical College, Vellore	India	April 2017 – December 2018	393	169 (43·0)	2017
	South Asia	India #2	Malankara Orthodox Syrian Church Medical College, Kolencherry	India	April 2017 – June 2018	318	124 (39·0)	Not introduced by end of 2018
	South Asia	India #3	Pandit Bhagwat Dayal Sharma Post Graduate Institute of Medical Sciences, Rohtak	India	April 2017 – December 2018	594	175 (29·5)	2016
	Southeast Asia and Oceania	Indonesia	Wates District Hospital / Dr. Sardjito Hospital, Yogyakarta	India	January 2017 – December 2018	322	171 (53·1)	Not introduced by end of 2018
	Southeast Asia and Oceania	Myanmar	Yangon Children's Hospital, Yangon	India	January 2017 – December 2018	1477	200 (13·5)	Not introduced by end of 2018
Western Pacific (WPR)	East Asia	China #1	Children's hospital of Suzhou, Jiangsu Province, Jiangsu	China	January 2017 – December 2018	516	169 (32·8)	Not introduced by end of 2018
	East Asia	China #2	Children's Hospital of Fuzhou City, Fuzhou	China	January 2017 – December 2018	617	181 (29·3)	Not introduced by end of 2018
	East Asia	China #3	People's Hospital of Lulong County, Jiangmen	China	January 2017 – December 2018	377	167 (44·3)	Not introduced by end of 2018
	Southeast Asia and Oceania	Fiji	Colonial War Memorial Hospital, Suva	Australia	January 2017 – December 2018	453	128 (28·3)	2013
	Southeast Asia and Oceania	Lao People's Democratic Republic	Mahosot Hospital, Vientiane	Australia	April 2017 – December 2018	448	136 (30·4)	Not introduced by end of 2018
	Southeast Asia and Oceania	Viet Nam	Hanoi National Hospital of Pediatrics, Hanoi	Australia	April 2017 – December 2018	1529	175 (11·4)	Not introduced by end of 2018

*Regional Reference Laboratories: Chinese Centers for Disease Control and Prevention, China; Christian Medical College, Vellore, India; Murdoch Children's Research Institute, Australia; Noguchi Memorial Institute for Medical Research, Ghana; Oswaldo Cruz Foundation (Fiocruz), Brazil; Republican Research and Practical Center for Epidemiology and Microbiology, Belarus; Sefako Makgatho Health Sciences University, South Africa; and U.S. Centers for Disease Control and Prevention.

Supplementary Table 2. Testing for established aetiologies of diarrhoea by qPCR on the TaqMan Array Card and corresponding gene target.

	Pathogen*	Gene
Viruses	Adenovirus 40/41	Fiber gene
	Astrovirus	Capsid
	Norovirus GII**	GII ORF1-2
	Rotavirus	*NSP3*
	Sapovirus	*RdRp*
Bacteria	Typical EPEC	*bfpA*
	ST-ETEC***	*STh* and *STp*
	Aeromonas	Aerolysin
	Campylobacter jejuni/coli	*cadF*
	Salmonella	*Ttr*
	Shigella/EIEC****	*ipaH*
	Vibrio cholera	*hlyA*
Protozoa	*Cryptosporidium*	18S rRNA
	Cyclospora cayetanensis	18S rRNA
	Cystoisospora belli	18S rRNA
	Entamoeba histolytica	18S rRNA
Controls	MS2	*MS2g1*
	PhHV	*gB*

*Targets for additional pathogens were included on the card but *a priori* were not included in the aetiology analysis because they were not associated with diarrhoea in both GEMS and MAL-ED[1,2], namely norovirus GI, enteroaggregative *E. coli* (EAEC), atypical enteropathogenic *E. coli* (aEPEC), heat labile toxin-producing *E. coli* (LT-ETEC), shiga toxin-producing *E. coli* (STEC), *H. pylori*, *Plesiomonas shigelloides*, *Enterocytozoon bieneusi*, *Encephalitozoon intestinalis*, *Giardia lamblia*, *Ancylostoma duodenale*, *Ascaris lumbricoides*, *Necator americanus*, *Strongyloides stercoralis*, and *Trichuris trichiura*.

** Because norovirus GI was not associated with diarrhoea in previous studies, norovirus GII is referred to as norovirus through this manuscript.

*** ST-ETEC was defined as the lower of the two quantification cycles for STh and STp. Because LT-ETEC was not associated with diarrhoea in previous studies, ST-ETEC is referred to as ETEC throughout this manuscript.

**** Based on previously reported evidence that the vast majority of *ipaH* detections in these settings represent *Shigella*, we refer to detection of *ipaH* as *Shigella* through this manuscript.

Supplementary Table 3. Pathogen-specific attributable fractions with 95% confidence intervals of diarrhoea hospitalizations by Global Pediatric Diarrhea Surveillance site in children less than 5 years of age, 2017-2018 (Figure 1).

Geographic Grouping	Surveillance Site	Rotavirus	*Shigella*	Norovirus	Adenovirus 40/41	Sapovirus	ETEC	*Cryptosporidium*	Astrovirus	*C. jejuni/coli*	*Salmonella*	tEPEC	*V. cholerae*
Central America	Honduras	17·8 (14·5, 19·0)	23·3 (12·6, 28·0)	11·2 (4·2, 20·2)	12·7 (4·2, 16·9)	2·5 (0·2, 4·0)	3·3 (1·3, 4·4)	2·2 (0·9, 2·9)	5·0 (1·3, 6·8)	3·9 (0·0, 5·5)	0·5 (0·2, 0·7)	0·0 (0·0, 2·6)	0·0 (0·0, 0·0)
	Nicaragua	17·2 (12·4, 19·9)	11·1 (8·1, 13·2)	11·4 (7·9, 13·0)	5·1 (0·8, 7·8)	3·9 (2·9, 4·7)	0·6 (0·4, 0·7)	0·8 (0·3, 1·1)	4·1 (2·6, 5·1)	0·6 (0·1, 1·0)	0·6 (0·0, 0·9)	0·3 (0·0, 0·5)	0·0 (0·0, 0·0)
South America	Bolivia	33·9 (25·7, 38·3)	8·9 (6·3, 10·6)	19·2 (9·5, 22·0)	1·7 (0·3, 2·9)	5·0 (1·7, 6·0)	6·7 (4·4, 8·1)	1·0 (0·4, 1·5)	0·9 (0·4, 1·1)	3·9 (1·5, 5·5)	0·0 (0·0, 0·0)	0·1 (0·0, 0·2)	0·0 (0·0, 0·0)
	Ecuador	5·5 (4·2, 6·1)	7·0 (6·0, 7·5)	25·4 (19·8, 28·5)	6·2 (2·6, 8·9)	5·7 (4·4, 6·6)	3·5 (2·5, 4·2)	0·9 (0·4, 1·2)	2·1 (1·3, 2·6)	4·0 (1·6, 5·7)	1·1 (0·0, 1·5)	0·5 (0·0, 1·0)	0·0 (0·0, 0·0)
	Peru	12·0 (8·9, 13·8)	13·9 (11·4, 15·5)	25·7 (20·9, 28·7)	6·0 (2·8, 7·8)	4·9 (3·8, 5·6)	1·9 (1·4, 2·3)	0·3 (0·1, 0·4)	2·0 (1·3, 2·6)	2·6 (0·9, 3·8)	1·1 (0·1, 1·7)	0·2 (0·0, 0·3)	0·0 (0·0, 0·0)
	Paraguay	7·0 (5·3, 7·9)	13·9 (11·0, 15·5)	15·8 (12·2, 17·9)	12·1 (4·5, 16·3)	2·0 (1·5, 2·2)	1·4 (1·0, 1·7)	0·8 (0·3, 1·1)	1·0 (0·6, 1·3)	1·4 (0·5, 2·0)	0·0 (0·0, 0·0)	0·7 (0·0, 1·4)	0·0 (0·0, 0·0)
Eastern Europe	Moldova	19·2 (15·1, 21·0)	6·8 (5·8, 7·2)	9·6 (7·6, 10·7)	7·4 (3·6, 9·2)	3·0 (2·2, 3·5)	1·5 (1·1, 1·8)	1·0 (0·5, 1·4)	0·5 (0·3, 0·6)	4·5 (1·6, 6·7)	2·3 (0·0, 3·3)	0·1 (0·0, 0·2)	0·0 (0·0, 0·0)
	Ukraine*	40·6 (32·6, 44·2)	0·0 (0·0, 0·0)	6·6 (5·7, 7·2)	1·4 (0·4, 2·1)	2·3 (1·7, 2·8)	0·1 (0·1, 0·1)	0·0 (0·0, 0·0)	0·9 (0·5, 1·1)	4·7 (1·9, 6·7)	1·8 (0·2, 2·6)	0·4 (0·0, 0·7)	0·0 (0·0, 0·0)
Central and Western Asia	Armenia	9·6 (7·3, 10·7)	6·8 (5·8, 7·3)	7·3 (5·6, 8·2)	2·2 (1·2, 2·5)	1·8 (1·3, 2·1)	3·7 (2·6, 4·5)	0·3 (0·2, 0·4)	0·8 (0·5, 1·1)	6·6 (2·4, 9·5)	0·7 (0·0, 0·9)	0·0 (0·0, 0·1)	0·0 (0·0, 0·0)
	Tajikistan	23·3 (18·4, 26·0)	13·7 (10·9, 15·5)	8·5 (6·0, 9·6)	3·5 (0·8, 4·8)	7·3 (5·4, 8·4)	4·1 (2·9, 4·9)	3·8 (1·9, 5·4)	4·6 (2·9, 5·5)	0·5 (0·1, 0·7)	0·0 (0·0, 0·0)	0·4 (0·0, 0·8)	0·0 (0·0, 0·0)
	Uzbekistan	10·2 (7·7, 11·2)	6·4 (5·2, 7·5)	16·3 (10·2, 19·4)	3·1 (0·0, 5·0)	4·9 (3·7, 5·7)	3·9 (2·6, 5·0)	0·9 (0·5, 1·3)	1·3 (0·8, 1·8)	0·2 (0·0, 0·4)	0·2 (0·0, 0·7)	0·5 (0·0, 1·0)	0·0 (0·0, 0·0)
West Africa	Benin*	39·1 (31·8, 40·6)	16·3 (8·0, 16·7)	2·9 (1·1, 5·9)	12·6 (8·5, 17·5)	1·3 (0·0, 2·5)	1·9 (0·7, 2·9)	3·3 (1·5, 3·9)	1·3 (0·2, 2·1)	3·5 (1·3, 4·8)	0·4 (0·1, 0·5)	0·0 (0·0, 7·2)	0·1 (0·0, 0·3)
	Cote d'Ivoire	9·0 (6·6, 9·9)	10·9 (8·6, 13·2)	0·7 (0·4, 1·0)	2·8 (0·0, 5·4)	2·6 (2·0, 3·1)	3·8 (2·6, 4·9)	2·2 (1·2, 2·9)	0·6 (0·4, 0·8)	1·2 (0·1, 2·6)	0·4 (0·0, 2·0)	2·1 (0·0, 4·3)	0·0 (0·0, 0·0)
	Ghana	32·1 (26·3, 35·0)	9·8 (3·5, 12·9)	5·8 (3·2, 6·8)	5·1 (0·0, 8·1)	2·6 (1·0, 3·4)	2·4 (1·4, 3·0)	5·9 (2·5, 9·0)	0·9 (0·5, 1·2)	2·4 (0·1, 4·1)	1·1 (0·0, 1·6)	1·7 (0·0, 3·4)	0·0 (0·0, 0·0)
	Nigeria*	53·7 (42·4, 58·7)	9·3 (3·4, 11·1)	4·7 (3·6, 5·4)	3·2 (0·9, 4·6)	5·1 (3·5, 6·2)	4·5 (3·2, 5·4)	3·4 (1·6, 4·8)	4·4 (2·6, 5·6)	0·2 (0·0, 0·3)	1·3 (0·1, 2·1)	0·4 (0·0, 0·7)	0·0 (0·0, 0·0)
East and Southern Africa	Ethiopia	12·9 (10·6, 14·1)	16·6 (12·9, 18·9)	5·4 (0·3, 7·9)	1·0 (0·0, 2·4)	3·9 (1·3, 5·1)	5·7 (3·9, 7·1)	5·8 (2·5, 7·7)	3·4 (1·9, 4·5)	2·0 (0·1, 4·1)	0·0 (0·0, 0·0)	1·1 (0·0, 2·2)	0·0 (0·0, 0·8)
	Madagascar	28·0 (23·5, 30·4)	12·9 (9·5, 14·7)	6·0 (2·5, 8·1)	8·3 (0·6, 12·3)	3·9 (1·5, 5·1)	4·8 (3·1, 5·6)	2·8 (1·2, 3·8)	1·5 (0·8, 1·8)	2·7 (0·0, 3·8)	0·9 (0·0, 1·5)	1·8 (0·0, 3·5)	0·0 (0·0, 0·0)
	Mauritius	11·7 (9·0, 13·1)	1·0 (0·7, 1·4)	16·9 (11·5, 19·0)	11·2 (0·0, 17·4)	4·8 (3·4, 6·0)	1·7 (1·2, 2·1)	0·1 (0·1, 0·2)	3·6 (2·3, 4·5)	2·5 (0·2, 3·8)	2·7 (0·0, 3·7)	0·9 (0·0, 1·8)	0·0 (0·0, 0·0)
	Uganda*	28·3 (21·2, 32·1)	4·6 (1·4, 5·5)	4·7 (3·6, 5·4)	6·5 (1·8, 9·4)	4·4 (2·9, 5·5)	5·5 (3·9, 6·8)	7·8 (3·0, 10·9)	1·1 (0·6, 1·5)	0·3 (0·1, 0·6)	0·7 (0·1, 1·1)	2·3 (0·0, 4·6)	1·0 (0·0, 1·0)
	Zambia #1	47·1 (35·2, 49·8)	9·4 (7·5, 10·7)	9·1 (4·1, 17·2)	12·7 (9·6, 17·6)	3·3 (0·0, 8·1)	10·2 (6·7, 12·9)	9·7 (4·5, 11·5)	2·5 (0·0, 4·2)	1·4 (0·2, 2·4)	1·6 (0·9, 1·7)	0·0 (0·0, 6·8)	0·6 (0·0, 0·6)
	Zambia #2	51·7 (38·4, 54·2)	15·5 (12·3, 17·7)	11·6 (5·0, 22·6)	22·1 (16·8, 31·8)	2·6 (0·0, 6·9)	8·9 (5·8, 11·5)	19·8 (10·4, 22·9)	0·8 (0·0, 1·3)	3·2 (0·5, 5·6)	1·8 (0·6, 2·2)	0·0 (0·0, 4·8)	0·0 (0·0, 0·0)
	Zimbabwe	35·5 (28·2, 39·0)	8·3 (5·4, 9·8)	9·2 (7·3, 10·5)	4·0 (0·4, 6·4)	4·8 (3·2, 6·1)	2·7 (1·9, 3·4)	1·9 (0·8, 2·8)	0·8 (0·5, 1·1)	1·9 (0·9, 2·7)	4·0 (1·0, 5·1)	0·1 (0·0, 0·2)	0·0 (0·0, 0·0)
South Asia	India #1	26·2 (22·4, 27·5)	15·1 (10·6, 18·6)	6·0 (2·9, 11·7)	24·2 (13·1, 29·4)	2·4 (0·0, 5·5)	1·3 (0·5, 2·1)	0·8 (0·4, 1·0)	1·4 (0·0, 2·3)	1·8 (0·0, 3·2)	2·9 (1·8, 3·3)	0·0 (0·0, 3·3)	0·1 (0·0, 0·2)
	India #2*	35·8 (30·9, 37·3)	8·5 (5·9, 10·6)	10·2 (4·5, 18·7)	15·1 (8·9, 18·8)	2·9 (0·0, 6·5)	1·3 (0·5, 2·1)	0·6 (0·2, 0·8)	1·1 (0·0, 2·0)	5·6 (0·0, 9·3)	1·6 (0·8, 1·7)	0·0 (0·0, 0·2)	0·0 (0·0, 0·0)
	India #3	13·3 (11·1, 14·1)	16·2 (11·0, 21·0)	4·2 (1·7, 8·8)	14·8 (8·4, 17·9)	4·4 (0·0, 9·7)	1·8 (0·6, 3·1)	4·5 (2·3, 5·4)	5·5 (0·0, 8·5)	2·3 (0·0, 3·9)	0·1 (0·0, 0·1)	0·0 (0·0, 4·1)	7·3 (1·4, 8·3)
Southeast Asia and Oceania	Fiji	12·8 (10·2, 14·0)	19·9 (15·8, 21·6)	14·5 (2·4, 16·0)	8·6 (0·0, 11·2)	5·4 (3·1, 6·4)	4·1 (3·0, 4·9)	1·0 (0·4, 1·5)	2·3 (1·4, 2·9)	2·0 (0·6, 3·0)	0·0 (0·0, 0·0)	2·5 (0·0, 4·8)	0·0 (0·0, 0·0)
	Indonesia*	29·0 (22·8, 32·0)	9·3 (7·0, 10·4)	7·6 (6·1, 8·3)	4·9 (1·6, 6·9)	1·4 (1·0, 1·7)	1·0 (0·7, 1·1)	0·2 (0·1, 0·2)	2·6 (1·7, 3·2)	3·0 (1·2, 4·2)	0·5 (0·1, 0·9)	0·0 (0·0, 0·0)	0·6 (0·0, 0·7)
	Laos*	35·3 (26·8, 39·1)	5·6 (4·5, 6·2)	12·8 (10·6, 14·0)	1·7 (0·9, 2·1)	2·8 (2·0, 3·4)	1·1 (0·9, 1·3)	1·5 (0·7, 2·1)	0·4 (0·2, 0·6)	2·2 (0·8, 3·3)	2·6 (0·1, 4·2)	0·2 (0·0, 0·4)	0·0 (0·0, 0·0)
	Myanmar*	64·3 (60·7, 66·1)	3·3 (2·6, 5·3)	4·9 (2·3, 6·8)	15·9 (12·7, 18·5)	1·7 (0·0, 3·9)	2·1 (0·9, 3·3)	1·1 (0·8, 1·3)	2·9 (0·0, 5·4)	0·0 (0·0, 2·8)	0·3 (0·2, 0·4)	0·0 (0·0, 0·0)	0·9 (0·4, 1·1)
	Vietnam*	16·3 (12·0, 18·2)	1·4 (1·1, 1·5)	19·9 (16·3, 21·9)	1·2 (0·5, 1·7)	3·8 (2·9, 4·4)	1·1 (0·8, 1·4)	0·1 (0·0, 0·1)	1·1 (0·6, 1·3)	3·3 (1·3, 4·4)	0·2 (0·0, 0·3)	0·0 (0·0, 0·0)	0·0 (0·0, 0·0)
East Asia	China #1*	21·4 (16·2, 24·9)	0·0 (0·0, 0·0)	5·6 (4·3, 6·5)	3·3 (0·9, 4·5)	1·0 (0·7, 1·2)	0·0 (0·0, 0·0)	0·0 (0·0, 0·0)	1·5 (0·8, 1·9)	0·4 (0·1, 0·6)	0·9 (0·0, 1·3)	0·0 (0·0, 0·1)	0·0 (0·0, 0·0)
	China #2*	21·7 (16·5, 24·0)	0·0 (0·0, 0·0)	8·8 (6·6, 9·8)	1·2 (0·4, 1·6)	1·9 (1·4, 2·2)	0·2 (0·1, 0·2)	0·0 (0·0, 0·0)	1·4 (0·9, 1·7)	1·0 (0·3, 1·5)	1·6 (0·0, 2·4)	0·0 (0·0, 0·0)	0·0 (0·0, 0·0)
	China #3*	41·1 (30·2, 45·9)	0·0 (0·0, 0·0)	25·6 (20·1, 28·2)	2·6 (0·6, 3·7)	1·0 (0·7, 1·3)	0·0 (0·0, 0·0)	0·0 (0·0, 0·0)	2·1 (1·2, 2·6)	0·7 (0·2, 1·1)	1·1 (0·0, 1·6)	0·3 (0·0, 0·7)	0·0 (0·0, 0·0)

Attributable fractions are expressed as a percent. *Rotavirus vaccine not introduced by 2017. tEPEC=typical enteropathogenic E. coli. ETEC=enterotoxigenic *E. coli*.

Cohen AL, *et al. BMJ Global Health* 2022; 7:e009548. doi: 10.1136/bmjgh-2022-009548

Supplementary Table 4. Pathogen-specific attributable fractions with 95% confidence intervals of diarrhoea hospitalizations both overall and by geographic grouping in children less than 5 years of age in countries participating in Global Pediatric Diarrhea Surveillance, 2017-2018 (Figure 2).

	Rotavirus	*Shigella*	Norovirus	Adenovirus 40/41	Sapovirus	ETEC	*Cryptosporidium*	Astrovirus	*C. jejuni/ C. coli*	*Salmonella*	tEPEC	*V. cholerae*
Overall	33·3 (27·7, 40·3)	9·7 (7·7, 11·6)	6·5 (5·4, 7·6)	5·5 (4·4, 6·7)	3·7 (2·9, 4·6)	3·7 (3·1, 4·4)	3·5 (2·7, 4·4)	2·7 (2·0, 3·5)	1·4 (0·9, 1·9)	1·0 (0·6, 1·3)	0·7 (0·4, 1·1)	0·4 (0·2, 0·5)
Central America	17·2 (12·8, 23·0)	19·2 (11·4, 28·1)	11·4 (5·7, 20·1)	10·0 (4·1, 15·0)	2·9 (1·3, 4·5)	2·3 (1·0, 3·7)	1·6 (0·8, 2·6)	4·5 (2·1, 6·8)	2·7 (0·2, 4·6)	0·5 (0·2, 0·8)	0·1 (0·0, 1·9)	0·0 (0·0, 0·0)
South America	15·4 (11·8, 19·9)	11·8 (9·3, 14·9)	22·2 (17·5, 27·9)	5·7 (3·6, 7·8)	4·5 (3·4, 5·6)	3·1 (2·3, 4·0)	0·6 (0·4, 0·8)	1·6 (1·1, 2·1)	2·8 (1·8, 3·9)	0·6 (0·2, 1·1)	0·2 (0·1, 0·4)	0·0 (0·0, 0·0)
Eastern Europe	37·0 (24·6, 52·8)	0·8 (0·6, 1·2)	6·8 (4·9, 9·6)	2·0 (1·1, 3·1)	2·3 (1·6, 3·4)	0·3 (0·2, 0·4)	0·1 (0·1, 0·2)	0·8 (0·5, 1·2)	4·5 (2·1, 7·5)	1·8 (0·4, 2·9)	0·3 (0·0, 0·7)	0·0 (0·0, 0·0)
Central and Western Asia	15·5 (11·6, 20·4)	9·5 (7·1, 12·5)	12·1 (8·2, 16·8)	3·1 (1·3, 4·8)	5·7 (4·2, 7·5)	3·9 (2·8, 5·3)	2·1 (1·2, 3·2)	2·7 (1·8, 3·7)	0·6 (0·3, 0·9)	0·1 (0·0, 0·4)	0·4 (0·1, 0·8)	0·0 (0·0, 0·0)
West Africa	44·5 (32·0, 60·1)	9·4 (5·0, 12·7)	4·1 (3·0, 5·6)	3·5 (1·8, 5·2)	4·3 (2·9, 6·1)	4·0 (2·8, 5·5)	3·4 (2·0, 5·0)	3·4 (2·0, 5·1)	0·6 (0·3, 0·9)	1·2 (0·3, 1·9)	0·7 (0·2, 1·2)	0·0 (0·0, 0·0)
East and Southern Africa	23·3 (19·6, 27·5)	12·2 (9·4, 15·6)	6·0 (3·6, 8·2)	5·0 (3·3, 6·6)	3·9 (2·6, 5·1)	5·5 (4·3, 6·9)	5·9 (4·1, 7·8)	2·2 (1·4, 3·0)	1·8 (0·9, 3·0)	0·7 (0·4, 1·0)	1·3 (0·5, 2·2)	0·2 (0·0, 0·6)
South Asia	24·5 (16·8, 35·2)	13·0 (7·8, 21·3)	6·6 (3·6, 13·9)	17·6 (10·6, 25·9)	3·0 (0·7, 7·6)	1·4 (0·8, 2·6)	1·9 (1·0, 2·9)	2·5 (0·5, 4·6)	3·1 (0·0, 5·9)	1·5 (0·9, 2·2)	0·0 (0·0, 2·3)	2·3 (0·1, 3·5)
Southeast Asia and Oceania	31·3 (24·7, 39·4)	6·3 (4·5, 8·6)	10·1 (7·8, 12·8)	5·6 (3·5, 7·7)	2·0 (1·5, 2·7)	1·2 (0·9, 1·6)	0·3 (0·3, 0·4)	2·2 (1·4, 3·1)	2·6 (1·5, 3·8)	0·5 (0·2, 0·7)	0·0 (0·0, 0·0)	0·5 (0·1, 0·7)
East Asia	27·3 (18·0, 40·6)	0·0 (0·0, 0·0)	13·0 (8·5, 19·2)	2·3 (0·9, 3·6)	1·3 (0·9, 1·9)	0·1 (0·0, 0·1)	0·0 (0·0, 0·0)	1·6 (1·0, 2·4)	0·7 (0·3, 1·1)	1·1 (0·2, 1·9)	0·1 (0·0, 0·3)	0·0 (0·0, 0·0)

Attributable fractions are expressed as a percent. tEPEC=typical enteropathogenic *E. coli*. ETEC=heat-stabile enterotoxigenic *E. coli*

Supplementary Table 5. Pathogen-specific attributable fractions with 95% confidence intervals of diarrhoea hospitalizations by age in children less than 5 years of age in countries participating in Global Pediatric Diarrhea Surveillance, 2017-2018 (Supplementary Figure 4).

	0-11 Months	12-23 Months	24-59 Months
Rotavirus	32.5 (26.6, 39.6)	33.5 (28.2, 39.8)	29.7 (25.1, 35.1)
Shigella	5.1 (3.8, 6.2)	12.9 (9.9, 15.7)	17.9 (14.0, 21.4)
Norovirus	6.4 (5.4, 7.6)	7.3 (6.1, 8.7)	3.9 (2.9, 4.9)
Adenovirus 40/41	5.2 (4.1, 6.4)	5.7 (4.6, 7.0)	6.7 (5.3, 8.3)
Sapovirus	3.3 (2.6, 4.1)	5.2 (4.0, 6.6)	1.4 (1.1, 1.9)
ETEC	2.7 (2.2, 3.4)	4.4 (3.6, 5.3)	4.3 (3.6, 5.2)
Cryptosporidium	2.4 (1.8, 3.0)	5.6 (4.1, 7.3)	3.3 (2.5, 4.1)
Astrovirus	1.7 (1.2, 2.2)	4.0 (2.8, 5.4)	1.6 (1.1, 2.0)
C. jejuni/C. coli	1.3 (0.8, 1.8)	1.7 (1.1, 2.4)	0.9 (0.6, 1.3)
Salmonella	0.5 (0.4, 0.7)	1.7 (0.6, 2.6)	1.0 (0.6, 1.5)
tEPEC	1.0 (0.5, 1.6)	0.6 (0.3, 1.0)	0.2 (0.1, 0.3)
V. cholerae	0.1 (0.1, 0.2)	0.3 (0.1, 0.7)	0.8 (0.1, 1.2)

Attributable fractions are expressed as a percent. tEPEC=typical enteropathogenic *E. coli*. ETEC=heat-stabile enterotoxigenic *E. coli*

Cohen AL, *et al. BMJ Global Health* 2022; 7:e009548. doi: 10.1136/bmjgh-2022-009548

BMJ Publishing Group Limited (BMJ) disclaims all liability and responsibility arising from any reliance placed on this supplemental material which has been supplied by the author(s)

Supplementary Table 6. Pathogen-specific attributable fractions with 95% confidence intervals of diarrhoea hospitalizations both overall and by World Health Organization region, stratified by introduction of rotavirus vaccine by 2017, in children less than 5 years of age in countries participating in Global Pediatric Diarrhea Surveillance, 2017-2018 (Figure 3).

	Rotavirus		*Shigella*		Norovirus		Adenovirus 40/41		Sapovirus		ETEC	
	RV not introduced	RV introduced	RV not introduced	RV introduced	RV not introduced	RV introduced	RV not introduced	RV introduced	RV not introduced	RV introduced	RV not introduced	RV introduced
Overall	42·1 (33·2, 53·4)	20·8 (18·0, 24·1)	7·0 (4·0, 9·4)	13·4 (10·9, 16·2)	7·0 (5·7, 8·6)	6·1 (4·2, 7·9)	5·1 (3·6, 6·5)	5·9 (4·4, 7·4)	3·8 (2·7, 5·1)	3·5 (2·5, 4·5)	3·1 (2·3, 4·2)	4·5 (3·6, 5·6)
African Region	48·3 (34·4, 65·5)	21·3 (18·1, 25·0)	8·4 (3·9, 11·9)	13·0 (10·4, 16·2)	4·5 (3·2, 6·1)	5·4 (3·3, 7·3)	3·9 (2·0, 5·7)	4·4 (2·9, 5·9)	4·8 (3·2, 6·7)	3·5 (2·3, 4·5)	4·5 (3·2, 6·1)	4·9 (3·8, 6·1)
Region for the Americas	NA	16·0 (12·9, 19·5)	NA	13·9 (11·0, 17·2)	NA	19·3 (15·3, 24·0)	NA	6·9 (4·8, 9·0)	NA	4·0 (3·1, 5·0)	NA	2·9 (2·2, 3·6)
European Region	39·2 (25·3, 58·3)	15·7 (11·9, 20·6)	0·0 (0·0, 0·0)	9·4 (7·1, 12·2)	6·4 (4·3, 9·6)	12·0 (8·3, 16·4)	1·3 (0·4, 2·4)	3·3 (1·6, 5·0)	2·2 (1·4, 3·5)	5·6 (4·2, 7·3)	0·1 (0·0, 0·1)	3·8 (2·7, 5·1)
South-East Asian Region	35·7 (28·3, 44·9)	19·2 (13·1, 27·6)	8·2 (5·9, 11·3)	15·4 (9·1, 25·1)	8·6 (5·1, 14·3)	4·9 (2·5, 10·9)	11·3 (7·4, 15·5)	19·0 (10·6, 28·3)	2·2 (0·5, 4·5)	3·2 (0·5, 8·0)	1·3 (0·8, 1·9)	1·5 (0·7, 2·8)
Western Pacific Region	25·3 (18·0, 35·8)	12·4 (8·1, 18·8)	0·4 (0·3, 0·5)	19·2 (12·7, 28·8)	14·2 (10·3, 19·3)	13·6 (2·4, 21·1)	2·1 (0·9, 3·1)	8·1 (0·0, 13·5)	1·8 (1·3, 2·4)	5·2 (2·8, 8·0)	0·3 (0·2, 0·4)	3·9 (2·4, 6·1)

Attributable fractions are expressed as a percent. RV=rotavirus vaccine, ETEC=enterotoxigenic *E. coli*

Supplementary Figure 1. Diarrhoea surveillance and qPCR testing by 3-month calendar quarters at each Global Pediatric Diarrhea Surveillance site, 2017-2018. Dark shading in each bar represents the subset of samples tested by qPCR, with a goal of 25 samples per 3-month quarter per year.

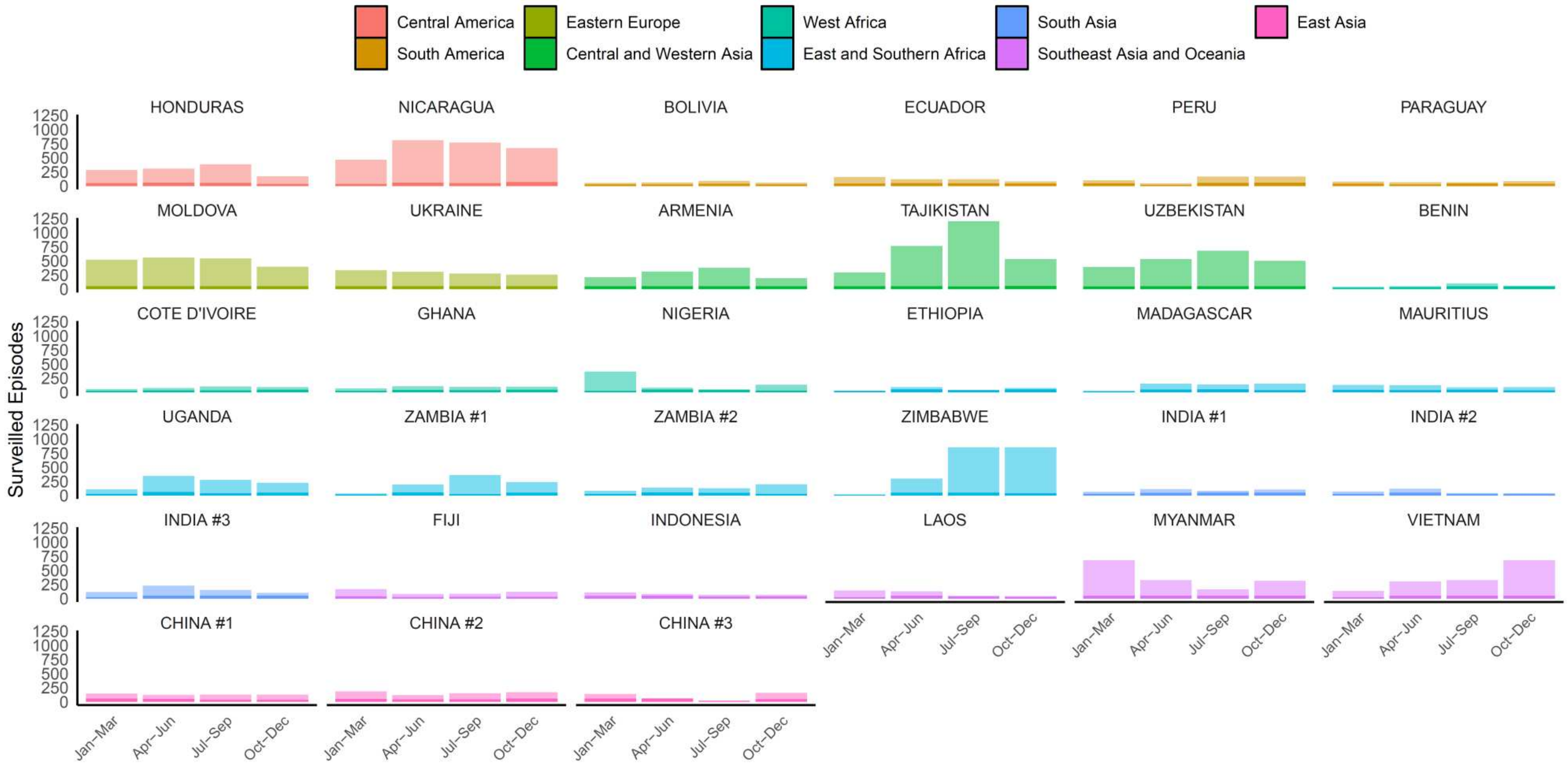

Supplementary Figure 2. Weighted prevalence by qPCR at quantification cycle cut-offs of 35 and 30 of diarrhoeal pathogens by Global Pediatric Diarrhea Surveillance site, 2017-2018. *Rotavirus vaccine not introduced by 2017. Cq = quantification cycle.

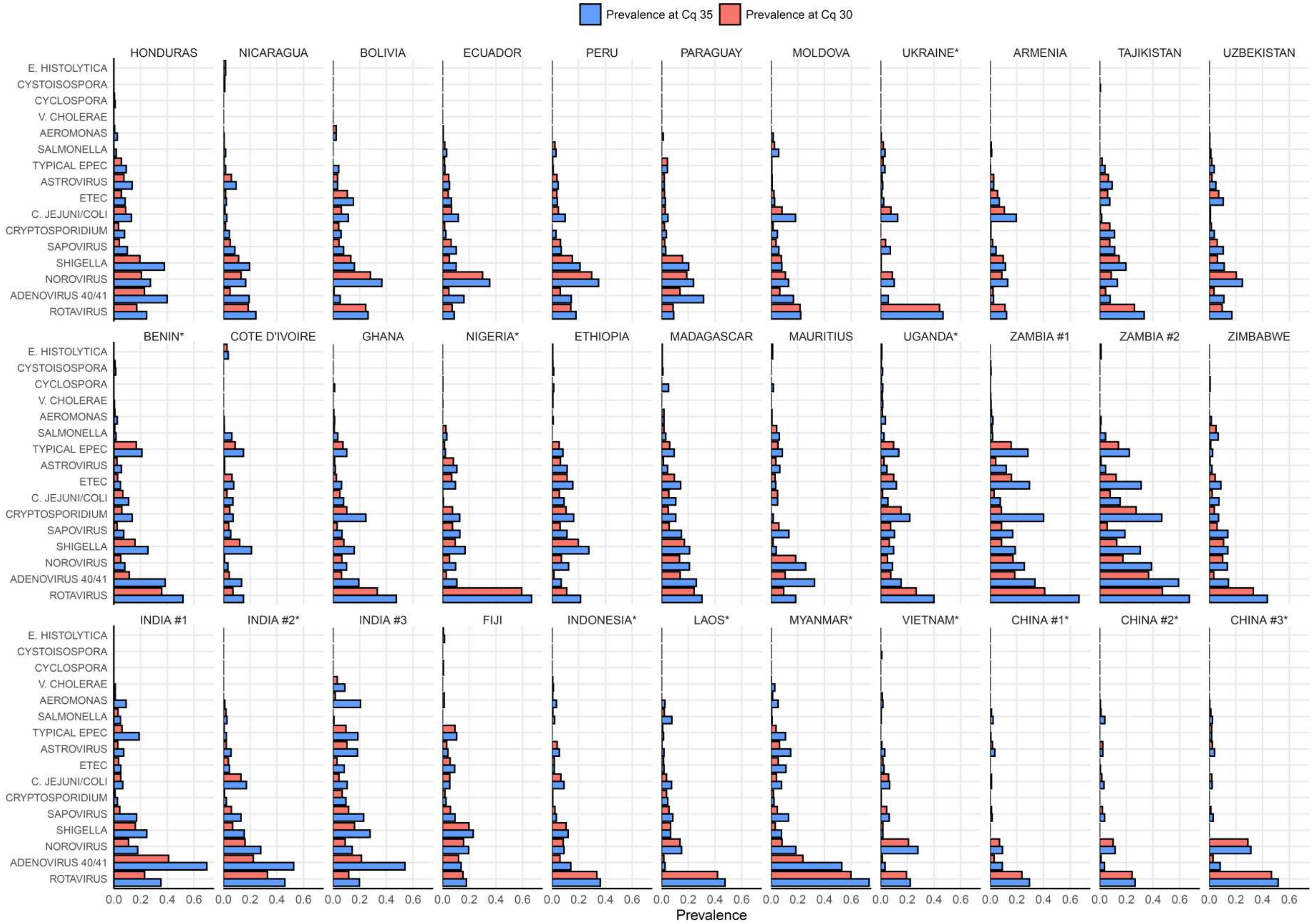

Cohen AL, *et al. BMJ Global Health* 2022; 7:e009548. doi: 10.1136/bmjgh-2022-009548

Supplementary Figure 3. Pathogen-specific attributable fractions by World Health Organization region and year. Estimates include all GPDS sites except Bolivia, for which testing was only performed in 2018. ETEC=enterotoxigenic *E. coli*. AFR = African Region; AMR = Region for the Americas; EUR = European Region; SEAR = South-East Asian Region; WPR = Western Pacific Region

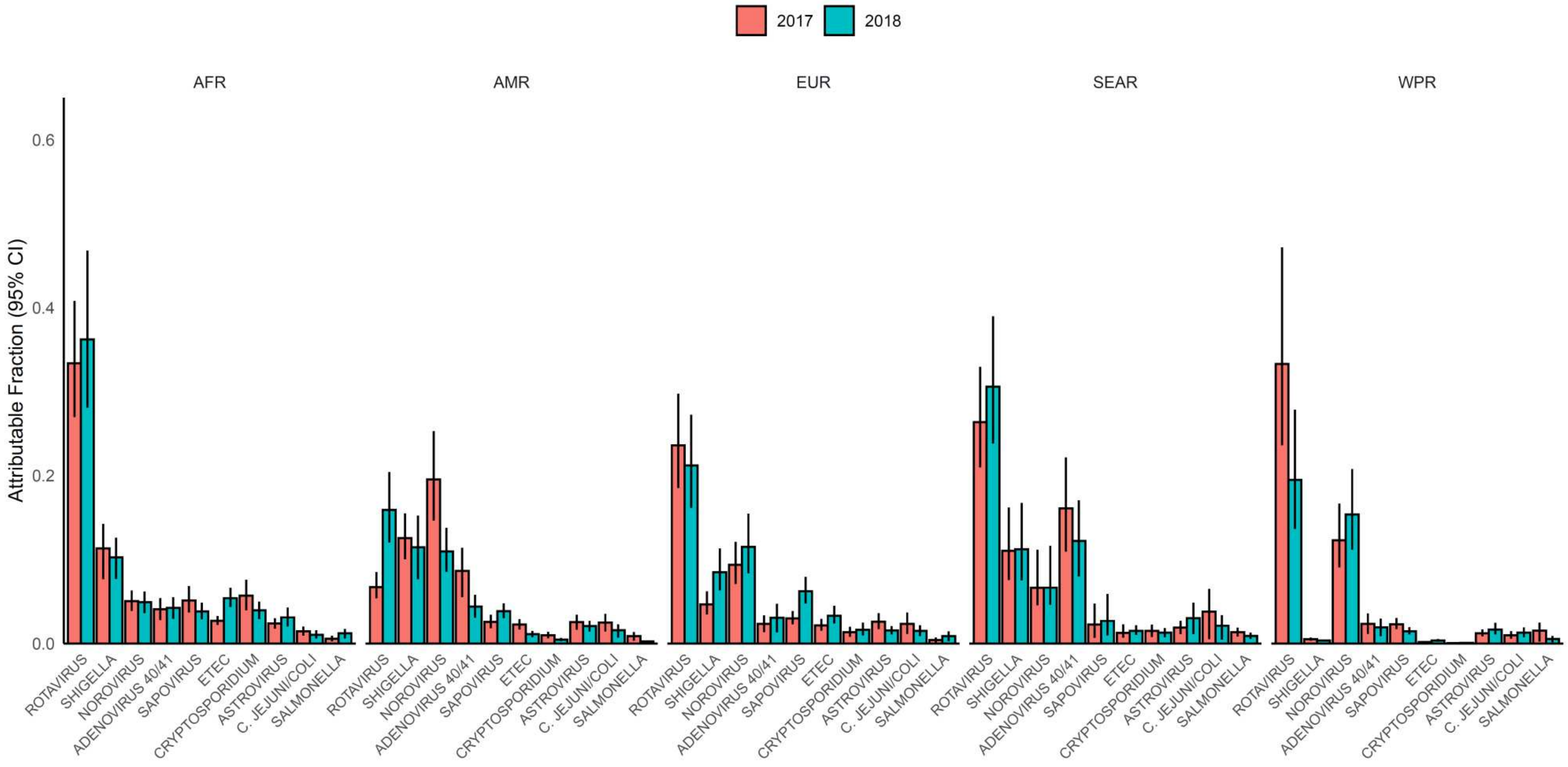

Supplementary Figure 4. Pathogen-specific attributable fractions by age. ETEC=enterotoxigenic *E. coli*.

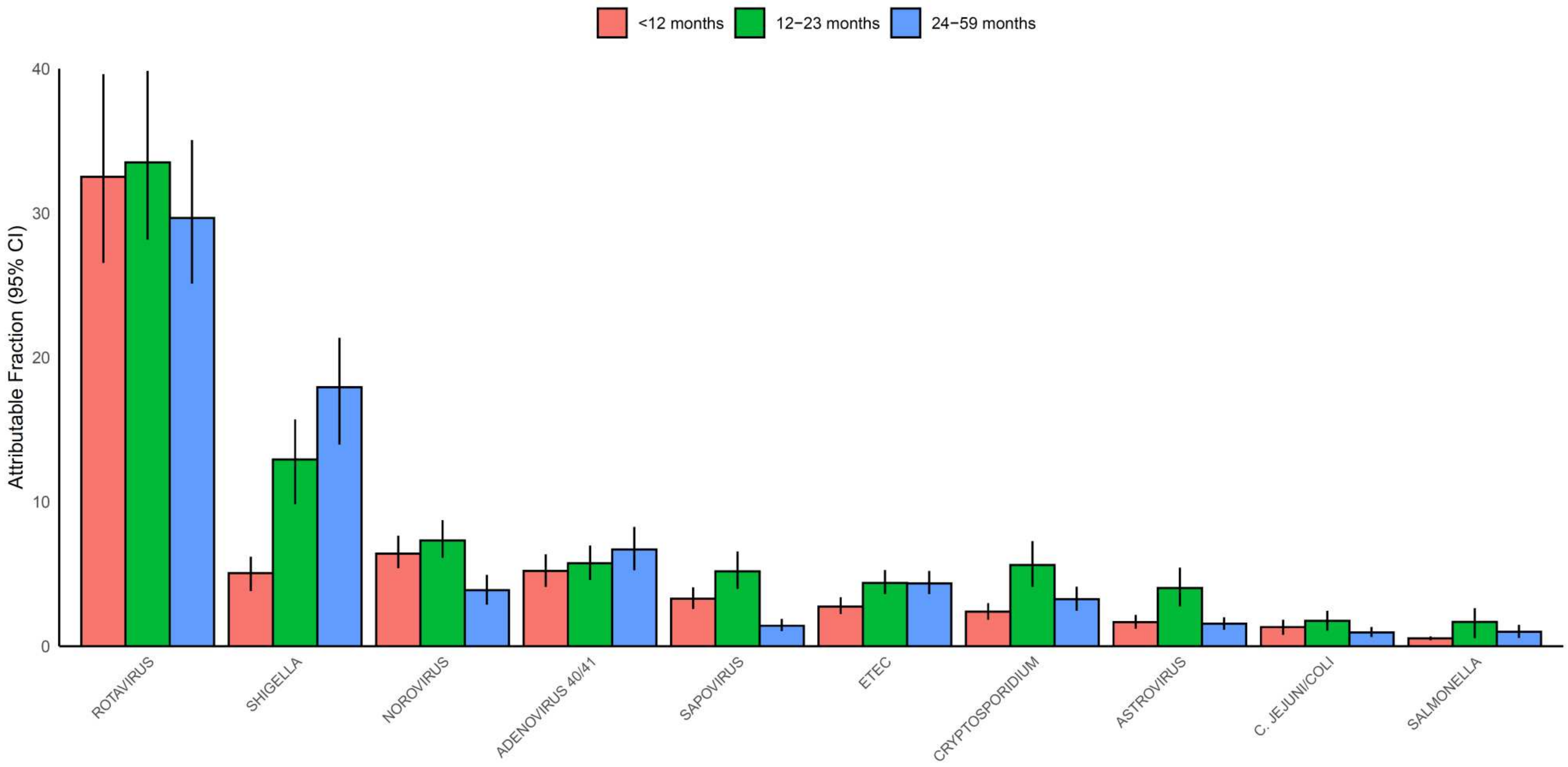

Supplementary Figure 5. Pathogen-specific attributable fractions by diarrhoeal syndrome. ETEC=enterotoxigenic *E. coli*.

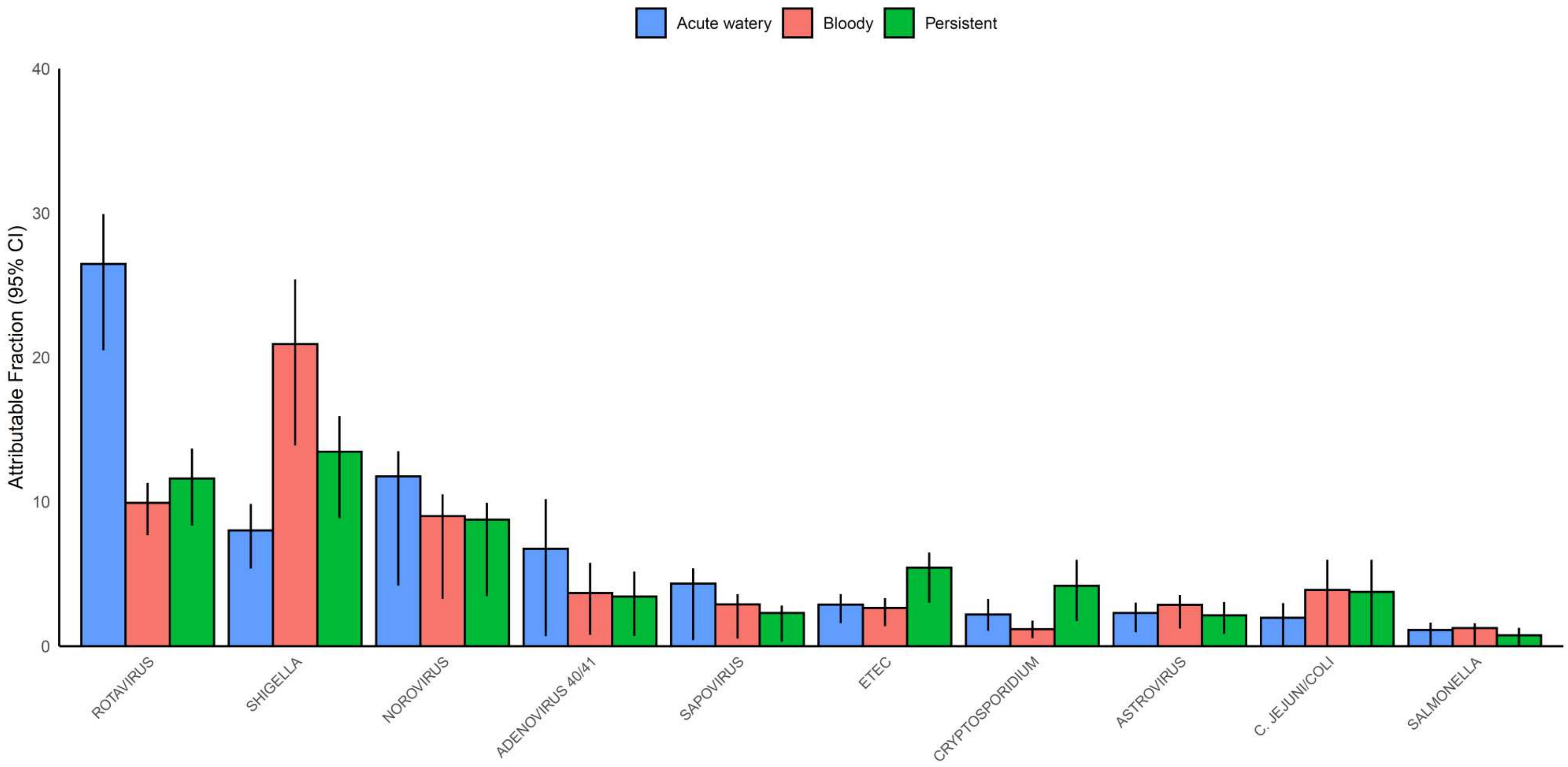

Cohen AL, *et al. BMJ Global Health* 2022; 7:e009548. doi: 10.1136/bmjgh-2022-009548

Supplementary Figure 6. Pathogen-specific attributable fractions by sex. ETEC=enterotoxigenic *E. coli*.

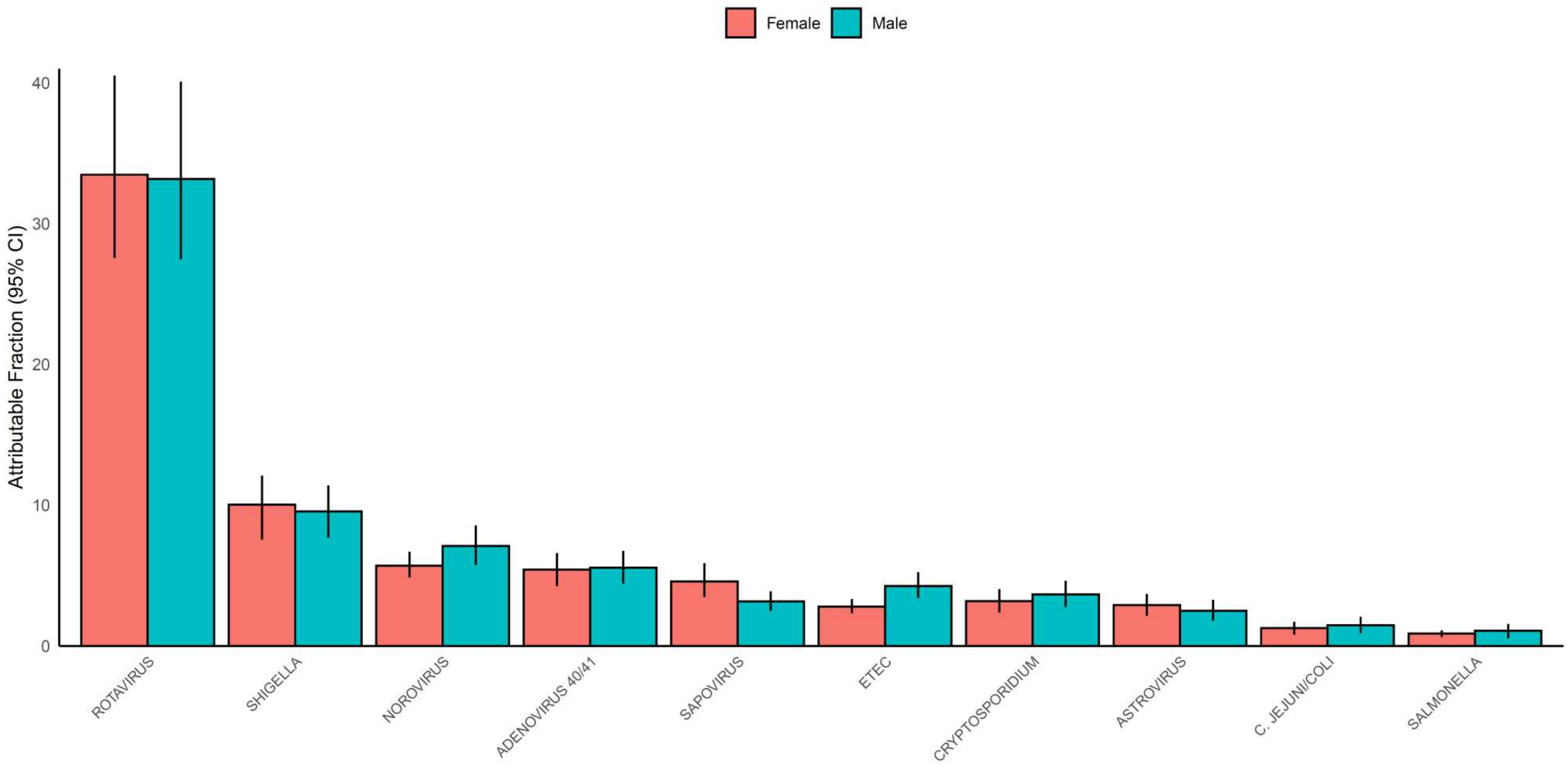

Supplementary Figure 7. Overall weighted prevalence of diarrhoeal pathogens in cases by qPCR at quantification cycle cut-offs of 35 and 30 and comparison to weighted attributable fractions in Global Pediatric Diarrhea Surveillance, 2017-2018. Cq = quantification cycle.

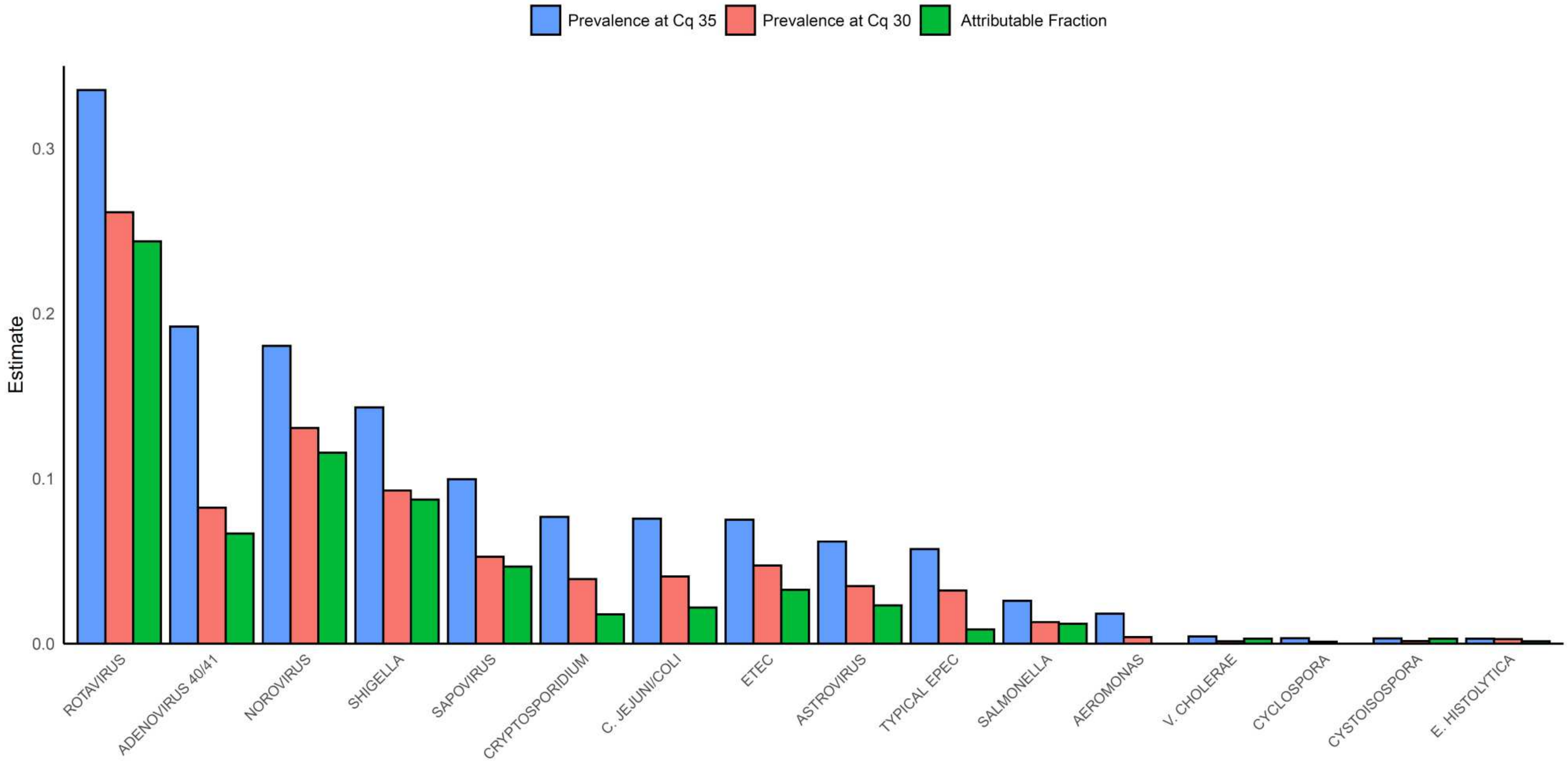

Supplementary Figure 8. Pathogen-specific attributable fractions estimated using even vs. optimized draws from the GEMS and MAL-ED models. In "even", Attributable Fractions were calculated using an even number of draws from each of the site-specific models from MAL-ED and GEMS. In "optimized", the number of draws per model for each GPDS site was weighted based on the similarity between the pathogen density in diarrhoea observed at that site and in each of the MAL-ED and GEMS sites. ETEC=enterotoxigenic *E. coli*.

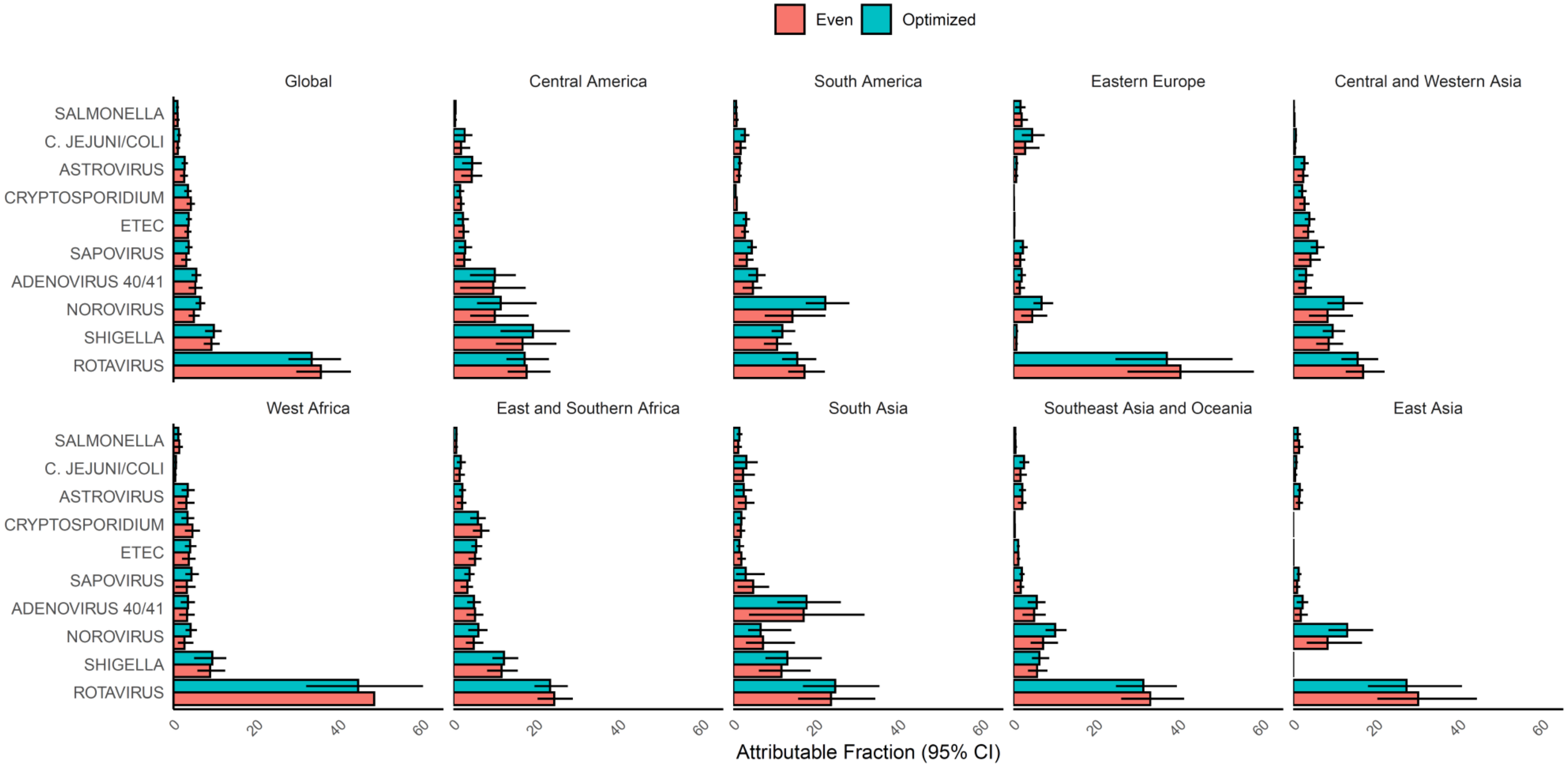

Cohen AL, *et al. BMJ Global Health* 2022; 7:e009548. doi: 10.1136/bmjgh-2022-009548

References

1. Liu J, Platts-Mills JA, Juma J, et al. Use of quantitative molecular diagnostic methods to identify causes of diarrhoea in children: a reanalysis of the GEMS case-control study. *Lancet* 2016; **388**(10051): 1291-301.

2. Platts-Mills JA, Liu J, Rogawski ET, et al. Use of quantitative molecular diagnostic methods to assess the aetiology, burden, and clinical characteristics of diarrhoea in children in low-resource settings: a reanalysis of the MAL-ED cohort study. *Lancet Glob Health* 2018; **6**(12): e1309-e18.

Cohen AL, *et al. BMJ Global Health* 2022; 7:e009548. doi: 10.1136/bmjgh-2022-009548
