# Supplementary material for: Aetiology and incidence of diarrhoea requiring hospitalisation in children under 5 years of age in 28 low-income and middle-income countries: findings from the Global Pediatric Diarrhea Surveillance network
Source: BMJ Glob Health. 2022 Sep 5;7(9):e009548. doi: 10.1136/bmjgh-2022-009548 (PMC9445824; doi:10.1136/bmjgh-2022-009548)
Supplement: Supplementary data [file bmjgh-2022-009548supp002.pdf]

Reflexivity Statement

Domain	Question	Response
Study conceptualization	1. How does this study address local research and policy priorities?	The burden of disease due to diarrhoea in LMICs is well established and remains a clear research priority to guide clinical interventions and inform vaccine policy, as has been evidenced by the substantial interest that local partners and institutions have had in supporting this work and maintaining surveillance.
	2. How were local researchers involved in study design?	The study design and protocol, including site selection, case definition, sample selection, and planned laboratory analyses, were developed collaboratively, starting at a global face-to-face meeting and continuing with regular in-person and virtual meetings with site and regional representatives.
Research management	3. How has funding been used to support the local research team(s)?	Funding was provided to sites for surveillance activities as well as to laboratories for sample testing and analysis.
Data acquisition and analysis	4. How are research staff who conducted data collection acknowledged?	The staff of the sentinel surveillance hospitals are explicitly acknowledged in the Acknowledgements section of the manuscript.
	5. How have members of the research partnership been provided with access to study data?	Study data has been made available to participating regions and sites.
	6. How were data used to develop analytical skills within the partnership?	Regional and global staff have worked closely with site and local staff to clean, analyze, interpret and use the data from surveillance, through local workshops and publications in peer-reviewed journals.
Data interpretation	7. How have research partners collaborated in interpreting study data?	Annual global and frequent regional face-to-face meetings, as well as more frequent virtual meetings, have been used to review results and incorporate feedback. Site visits to individual countries were also conducted every 1-2 years when possible.
Drafting and revising for intellectual content	8. How were research partners supported to develop writing skills?	All authors in the participating countries contributed to the drafting and revision of the manuscript. Additional region- and site-specific analyses are planned that will be led by research partners.
	9. How will research products be shared to address local needs?	Country-level reports are shared with each surveillance site.
Authorship	10. How is the leadership, contribution and ownership of this work by LMIC researchers recognised within the authorship?	Authors from all participating regions and reference labs are included; the majority of authors and collaborators are from LMICs. One of the first authors and the senior author represent WHO, and the senior author is originally from Lebanon.
	11. How have early career researchers across the partnership been included within the authorship team?	We have intentionally supported and included early career researchers from LMICs, especially through the Regional Reference Laboratories and WHO. Depending on the definition, we would estimate 5-10 of the authors.
	12. How has gender balance been addressed within the authorship?	23 authors identify as male and 22 as female, including a female senior author
Training	13. How has the project contributed to training of LMIC researchers?	In addition to site visits and trainings, we have conducted frequent regional laboratory and surveillance trainings with LMIC site representatives.
Infrastructure	14. How has the project contributed to improvements in local infrastructure?	The site surveillance and regional reference laboratory infrastructure has received substantial development and capacitation by participation in the Global Rotavirus and Pediatric Diarrhea Surveillance Networks.
	15. What safeguarding procedures were used to protect local study participants and researchers?	All analyses were performed on de-identified data.
